# Rhizosphere competent inoculants modulate the apple root–associated microbiome and plant phytoalexins

**DOI:** 10.1007/s00253-024-13181-8

**Published:** 2024-05-27

**Authors:** Kristin Hauschild, Nils Orth, Benye Liu, Adriana Giongo, Silvia Gschwendtner, Ludger Beerhues, Michael Schloter, Doris Vetterlein, Traud Winkelmann, Kornelia Smalla

**Affiliations:** 1 Institute for Epidemiology and Pathogen Diagnostics, Julius Kühn Institute, Federal Research Centre for Cultivated Plants, Braunschweig, Germany; 2 Institute of Horticultural Production Systems, Leibniz University Hannover, Hannover, Germany; 3 Institute of Pharmaceutical Biology, Technische Universität Braunschweig, Braunschweig, Germany; 4 Research Unit Comparative Microbiome Analysis, Helmholtz Centre Munich, Munich, Germany; 5 Department of Soil System Science, Helmholtz Centre for Environmental Research, Halle/Saale, Germany

**Keywords:** Apple replant disease, *Bacillus velezensis* FZB42, *Pseudomonas* sp. RU47, Rhizoplane, Root endosphere, Soil

## Abstract

**Abstract:**

Modulating the soil microbiome by applying microbial inoculants has gained increasing attention as eco-friendly option to improve soil disease suppressiveness. Currently, studies unraveling the interplay of inoculants, root-associated microbiome, and plant response are lacking for apple trees. Here, we provide insights into the ability of *Bacillus velezensis* FZB42 or *Pseudomonas* sp. RU47 to colonize apple root-associated microhabitats and to modulate their microbiome. We applied the two strains to apple plants grown in soils from the same site either affected by apple replant disease (ARD) or not (grass), screened their establishment by selective plating, and measured phytoalexins in roots 3, 16, and 28 days post inoculation (dpi). Sequencing of *16S rRNA* gene and ITS fragments amplified from DNA extracted 28 dpi from different microhabitat samples revealed significant inoculation effects on fungal β-diversity in root-affected soil and rhizoplane. Interestingly, only in ARD soil, most abundant bacterial amplicon sequence variants (ASVs) changed significantly in relative abundance. Relative abundances of ASVs affiliated with *Enterobacteriaceae* were higher in rhizoplane of apple grown in ARD soil and reduced by both inoculants. Bacterial communities in the root endosphere were not affected by the inoculants but their presence was indicated. Interestingly and previously unobserved, apple plants responded to the inoculants with increased phytoalexin content in roots, more pronounced in grass than ARD soil. Altogether, our results indicate that FZB42 and RU47 were rhizosphere competent, modulated the root-associated microbiome, and were perceived by the apple plants, which could make them interesting candidates for an eco-friendly mitigation strategy of ARD.

**Key points:**

• *Rhizosphere competent inoculants modulated the microbiome (mainly fungi)*

• *Inoculants reduced relative abundance of Enterobacteriaceae in the ARD rhizoplane*

• *Inoculants increased phytoalexin content in roots, stronger in grass than ARD soil*

**Supplementary information:**

The online version contains supplementary material available at 10.1007/s00253-024-13181-8.

## Introduction

Apple replant disease (ARD) is a phenomenon occurring in tree nurseries and apple orchards worldwide. Replanting apples on the same soil as previous apple cultures leads to severe disease symptoms, resulting in reduced plant growth and yield losses (Mazzola and Manici 2012; Winkelmann et al. 2019; Somera and Mazzola 2022). Despite decades of research trying to elucidate the etiology of ARD, the disease is still not fully understood. ARD is hypothesized to be caused by a dysbiosis of microorganisms in the soil that was previously grown with apple plants (Winkelmann et al. 2019). Typical disease symptoms are root blackening, reduced root branching, and root infections with plant pathogens (Caruso et al. 1989; Grunewaldt-Stöcker et al. 2019, 2021). The symptoms are associated with a strong plant stress response to ARD-affected soil in which plant defense molecules like phenolic compounds, especially phytoalexins, are accumulated in the roots and differentially exuded to the soil (Henfrey et al. 2015; Weiß et al. 2017; Busnena et al. 2021; Reim et al. 2022). Several studies revealed that the rhizosphere microbial community composition of apple plants grown in ARD-affected soils is distinct from that found in plants grown in soil from the same site but with no history of apple cultivation (Sun et al. 2014; Franke-Whittle et al. 2015; Yim et al. 2015; Tilston et al. 2018; Balbín-Suárez et al. 2020, 2021). For the root endosphere, species belonging to the genera *Streptomyces*, *Ilyonectria*, *Thelonectria*, *Rhizoctonia*, or *Pythium* were often reported in higher densities in ARD-affected roots and therefore considered to contribute to the ARD causing complex (Manici et al. 2013, 2018; Popp et al. 2020; Mahnkopp-Dirks et al. 2022). However, the abundance, diversity, and even presence or absence of taxa related to ARD vary highly between regions and orchards (Mazzola et al. 2002; Tewoldemedhin et al. 2011; Manici et al. 2013).

The predominant measure to counteract ARD remains chemical soil fumigation (Mai and Abawi 1978; Willett et al. 1994; Yim et al. 2013). However, due to their toxicity and environmental harm, the application of these chemicals was prohibited in most European countries (Ruzo 2006; Porter et al. 2010; Prashar and Shah 2016). In Germany, the use of the pesticide Basamid® granular, mainly used for chemical mitigation of ARD, will be prohibited from June 2024 (Federal Office of Consumer Protection and Food Safety 2020). Thus, there is an urgent need to develop sustainable alternative treatment options to counteract ARD. Over the past years, alternatives to soil fumigation like spatial reorganization of planting in the orchards, biofumigation, or breeding of tolerant rootstocks have been evaluated (Leinfelder and Merwin 2006; Rumberger et al. 2007; Yim et al. 2016, 2017). Surprisingly, reports on the effects of microbial inoculants on the rhizosphere microbiome and the response of the plant in ARD-affected soils are rare (Somera and Mazzola 2022). Only a few studies investigated the effect of beneficial bacteria, mainly members of the genus *Bacillus*, with regard to ARD, focusing on plant growth but typically not considering effects on the microbiome (Utkhede et al. 2001; Karlidag et al. 2007; Duan et al. 2021, 2022a, 2022b). Currently, reports on effects of bacterial inoculants on the microbial community composition and microbiome modulation in apple are missing. Also, the molecular plant response to inoculation has not been investigated before.

Isolates belonging to *Bacillus* spp. have several traits that make them promising candidates for biostimulation. The Gram-positive bacterium is a spore-former allowing survival under extreme conditions (Santoyo et al. 2012; Shafi et al. 2017). *Bacillus* strains are easy to ferment and harvest as spores at a large-scale, enabling their production as a biocontrol product (Fira et al. 2018). *Bacillus velezensis* FZB42 (formerly classified as *Bacillus amyloliquefaciens*) is considered “the Gram-positive model strain for plant growth promotion and biocontrol” (Fan et al. 2018). This strain and its potential for plant growth promotion was studied for many years, and the genome sequence is available (Chen et al. 2007). Also, *Pseudomonas* commonly occur in the rhizosphere and characteristics like the ability to colonize and proliferate in the rhizosphere, the effective use of root exudates, and the potential to suppress soil-borne pathogens make also isolates belonging to *Pseudomonas* spp. promising candidates for biostimulation (Weller et al. 2002; Preston 2004; Weller 2007; Santoyo et al. 2012). The strain *Pseudomonas* sp. RU47 (previously *Pseudomonas jessenii* RU47) was isolated from a disease suppressive soil (Adesina et al. 2007) and the analyses of its genome revealed several genes coding for plant beneficial traits (Kuzmanović et al. 2018).

Previous studies using FZB42 or RU47 mainly focused on a variety of annual plants, including tomato, cucumber, lettuce, cotton, or tobacco (Grosch et al. 1999; Yao et al. 2006; Gül et al. 2008; Adesina et al. 2009; Wang et al. 2009; Chowdhury et al. 2013; Windisch et al. 2017; Schreiter et al. 2018; Eltlbany et al. 2019). PGPR traits of FZB42, like the secretion of secondary metabolites and production of hydrolytic enzymes, the stimulation of induced systemic resistance and positive effects on the microbiome, have been reviewed extensively (Chowdhury et al. 2015; Fan et al. 2018; Amaresan et al. 2020). The potential of RU47 to establish in the rhizosphere and to enhance plant growth or suppress plant pathogens was shown for potato, tomato, or lettuce (Adesina et al. 2009; Schreiter et al. 2018; Eltlbany et al. 2019). The strain’s capability to solubilize phosphate and to produce indole-3-acetic acid, siderophores, HCN, and protease was confirmed by *in vitro* testing (Adesina et al. 2009; Kuzmanović et al. 2018). Successful plant growth promotion or biostimulation require the establishment of the inoculants in the rhizosphere of the targeted host plant. Recently, Behr et al. (2023) showed in field trials that RU47 was capable to survive in the rhizosphere of winter rye over one winter period. Berg et al. (2021) stated that the effective colonization of inoculants *in situ* is one of the essential steps for successful interaction with the plant and the native microbiome. Although both strains, FZB42 and RU47, are well characterized and were successfully used as inoculants on several cultivated plants, to our knowledge, their interplay with apple plants and their native microbiome has not been investigated before.

In this study, our objective was to elucidate the colonization potential of strains FZB42 and RU47 in root-affected soil (RA) and on the rhizoplane (RP) of apple plants. Additionally, we sought to understand their impact on the microbiome within the RA, RP, and the root endosphere (RE). We hypothesized that the plant response to the inoculation depends on the inoculants’ rhizosphere competence and the degree of microbiome modulation. Therefore, we determined the inoculants’ colony forming units (CFU) counts 3, 16, or 28 days post inoculation (dpi) of young apple plants grown under greenhouse conditions in soil from the same site affected by ARD or not (grass soils). We analyzed the microbiome in the different microhabitats RA, RP, and RE by a DNA-based meta-barcoding approach. Plant response to the inoculants was analyzed by investigating root phytoalexin content and morphology.

## Materials and methods

### Soils

Topsoils (0–20 cm) were collected from a site in Ellerhoop, Germany (53° 42′ 51.71″ N, 9° 46′ 12.16″ E), in May 2020. Soil from this site was classified as Endostagnic Luvisol (FAO and ITPS 2015). Details on soil texture and abiotic soil properties were reported previously (Mahnkopp et al. 2018). The site was established in 2009 with ARD plots (*n* = 4) by replanting apple rootstocks ‘Bittenfelder Sämling’ every other year (ARD soil) and grass plots (*n* = 4) with no apple cultivation history (Mahnkopp et al. 2018). Soils from both variants were taken as pooled samples from all plots and homogenized by sieving through a 2-mm mesh. For the greenhouse experiment, both soils were mixed with 50% (v/v) sterilized sand and fertilized with 2 g L^-1^ Osmocote exact 3–4 M (16% *N* + 9% P_2_O_5_ + 12% K_2_O + 2% MgO, ICL Deutschland, Nordhorn, Germany). These mixtures will further be referred to as ARD and grass soils.

### Bacterial inoculant strains

The rifampicin-resistant strain *Bacillus velezensis* FZB42 (DSMZ, Braunschweig, Germany, No.: DSM23117) was provided as a ready-to-use spore suspension of 6.7 × 10^9^ CFU mL^−1^ and derived from the commercial product Rhizovital (ABiTEP GmbH, Berlin, Germany). The cultivation was performed on Reasoners’ 2 agar (R2A, Merck Millipore, Burlington, MA, USA) supplemented with rifampicin (75 µg mL^−1^) and cycloheximide (100 µg mL^−1^) (thereafter called medium “MB”). Strain *Pseudomonas* sp. RU47 (DSMZ, No.: DSM117411) was obtained from our laboratory strain collection and was resistant to rifampicin, tetracycline, chloramphenicol, and ampicillin. Initial cultivation of the strain was carried out on King’s B agar (KB, Carl Roth, Karlsruhe, Germany) supplemented with rifampicin (75 µg mL^−1^), ampicillin (100 µg mL^−1^), chloramphenicol (30 µg mL^−1^), tetracycline (10 µg mL^−1^), and cycloheximide (100 µg mL^−1^) (thereafter called medium “MP”). Agar plates for both strains were incubated at 28 °C for 48 h until single colonies were observed. Overnight cultures of RU47 were grown in Luria Bertani broth (LB, Carl Roth, Karlsruhe, Germany) supplemented with the aforementioned antibiotics at 28 °C and 150 rpm on a shaker.

### Plant material and greenhouse experiment

**Fig. 1:**
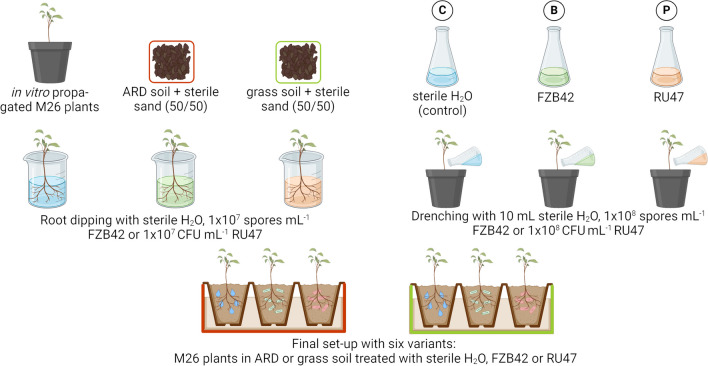
Scheme of the experimental set-up of the performed greenhouse trial. Apple plants of rootstock M26 were *in vitro* propagated and potted in 400 mL of ARD or grass soil, each mixed with 50% sterile sand. Before planting, plants were inoculated by dipping roots in the inoculation suspensions: sterile H_2_O (C), 1 × 10^7^ spores mL^−1^ *Bacillus velezensis* FZB42 (B) or 1 × 10^7 ^ CFU mL^−1^  of *Pseudomonas* sp. RU47 (P). After planting, each plant was drenched with 10 mL of C, B, or P with a concentration of B and P of 1 × 10^8^ spores mL^−1^ or CFU mL^−1^, respectively. Finally, plants of six differently treated variants were set up and grown for 3, 16, or 28 days. Created with biorender.com

Plant material of the ARD-susceptible rootstock genotype M26 was propagated *in vitro* as described earlier (Rohr et al. 2020). In brief, shoot cultures were multiplied on MS (Murashige and Skoog 1962) medium containing 3% (w/v) sucrose, 4.4 µM 6-benzylaminopurine, and 0.5 µM indole-3-butyric acid (IBA). For rooting, single shoots were transferred to rooting medium (1/2 MS with 3% (w/v) sucrose and 4.92 µM IBA) for 3 weeks, before the plants were acclimatized in a commercial peat substrate (Steckmedium, Klasmann-Deilmann GmbH, Geeste, Germany). Forty-three-day-old plants (counting from transfer of the plants into the substrate) were used for the greenhouse experiment. At set-up, the substrate was carefully removed around the roots. Both bacterial strains, FZB42 and RU47, were inoculated by root-dipping followed by drenching around the stem after planting in the soils. Root dipping and drenching with sterile tap water without inoculum served as control (treatment C). For root dipping, inoculation suspensions were prepared. For FZB42, the provided spore suspension was mixed thoroughly and diluted in sterile tap water to 1 × 10^7^ spores mL^−1^ (treatment B). For RU47, overnight cultures were grown and pelleted at 4000 *g* for 20 min at 4 °C. The cell pellet was washed in 50 mL sterile 0.85% NaCl twice, and the centrifugation was repeated. Finally, the cells were diluted to 1 × 10^7^ CFU mL^−1^ in sterile tap water (treatment P). The roots of plants to be treated were placed in 250 mL of inoculation suspension for 30 min. After root dipping, each plant was transferred into a pot with 400 mL soil (1.2 g mL^−1^) and subsequently subjected to drenching with 10 mL sterile H_2_O (C), 10 mL 1 × 10^8^ spores mL^−1^ of FZB42 or 10 mL 1 × 10^8^ CFU mL^−1^ of RU47. Pots were placed in trays laid out with fleece mats facilitating steady watering from below. Plants were irrigated every other day by evenly wetting the fleece mats. Cultivation occurred from June 06, 2020, until July 07, 2020, in a greenhouse chamber at a mean temperature of 19.9 ± 1.8/18.4 ± 0.9 °C (day/night) with a 16-h photoperiod. The average relative humidity during the experiment was 71.2 ± 10.9%. If the photosynthetic active radiation (PAR) was below 182 µmol m^2^ s^−1^ during the photoperiod, additional light was supplied by high-pressure sodium lamps (MASTER SON-T PIA Plus, Phillips Lightning, Eindhoven, Netherlands). The two soils and three treatments led to six differently treated variants: M26 grown in ARD or grass soil and treated with C, B, or P (Fig. 1).

### Sample collection and processing

**Fig. 2:**
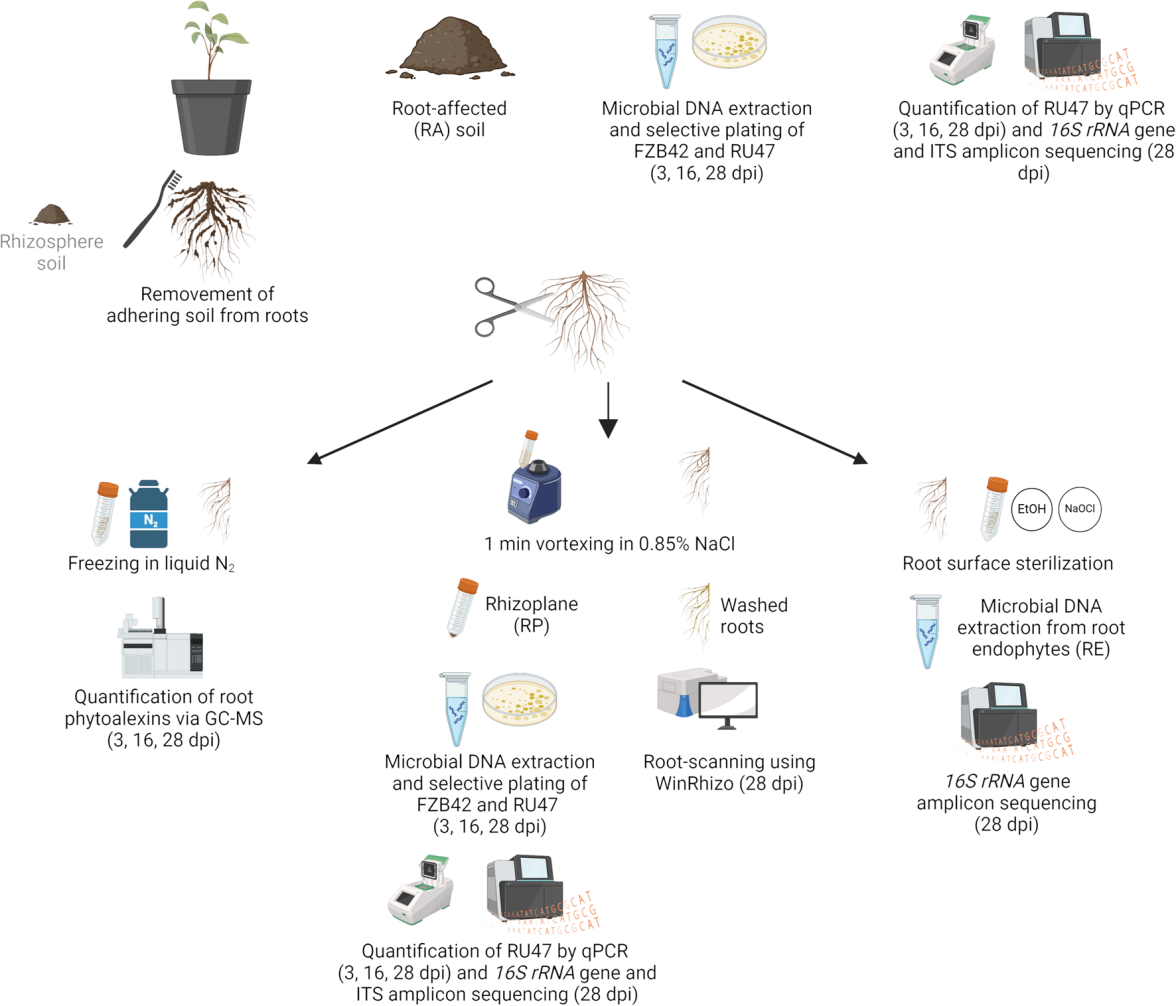
Scheme of destructive samplings and separation of materials to be analyzed 3, 16, and 28 days post inoculation (dpi). FZB42: *Bacillus velezensis* FZB42; RU47: *Pseudomonas* sp. RU47. Created with Biorender.com

Destructive samplings of RA, RP, and roots for the extraction of phytoalexins (PA) were conducted with four replicates per variant 3, 16, and 28 dpi (Fig. 2). Twenty-eight dpi, RE and root morphology were analyzed using six and seven replicates per variant, respectively. Plants were carefully taken out of the pots. The soil remaining in the pots (RA) was mixed well, and 1 g was resuspended in 1:10 (w/v) 0.85% NaCl and vortexed for 1 min. Root systems were separated from the shoot using sterile scalpels and split in half. One half of the root system was rinsed gently under water, dried on a paper towel, immediately frozen in liquid nitrogen, and stored at − 80 °C until PA extraction. From the second half, loosely adhering soil was gently removed from the roots using toothbrushes to detach the rhizosphere which was not analyzed further. To obtain RP, the brushed roots were cut in 3–4 cm pieces and RP was detached by vortexing in 1:10 (w/v) 0.85% NaCl for 1 min. RA and RP suspensions were used for selective plating within 2 h after obtaining the solution. For subsequent molecular analysis, the remaining RA and RP solutions were centrifuged at 4000 *g* for 20 min at 4 °C and the pellets were stored at − 20 °C until microbial community DNA was extracted. Twenty-eight dpi, root systems were split into three parts: (I) for PA extraction, (II) for the harvest of RP and subsequent storage of roots in 50% alcohol solution (i.e. diluted Rotisol®) until analysis of root morphology (four split root systems and three additional entire root systems), and (III) for the analysis of RE (four split root systems and two additional root systems). RE samples were rinsed under tap water and stored in 50 mL reaction tubes in the dark (100% humidity, 4 °C). The next day, roots were surface-disinfected as described in Mahnkopp-Dirks et al. (2021). The surface-disinfected roots were cut into approximately 1 cm pieces and dried for a short period on sterile filter paper under a laminar flow. Approximately 60–100 mg were transferred to a 2 mL reaction tube, frozen in liquid nitrogen and stored at − 80 °C until DNA extraction.

### Phytoalexin extraction and quantification by GC–MS

Aliquots of the root samples described above were lyophilized and homogenized to a fine powder (29 Hz, 1 min; Mixer Mill MM400, Retsch, Haan, Germany). The protocols used for PA extraction as well as detection and quantification by GC–MS after silylation are well-established techniques, which were described previously (Weiß et al. 2017; Balbín-Suárez et al. 2021; Busnena et al. 2021). Briefly, the root powder was extracted with 1 mL methanol containing 25 µg 4-hydroxybiphenyl (internal standard for relative quantification) by vigorous vortexing (2700 rpm, 20 min, Vortex Genie2, Scientific Industries, Bohemia, NY, USA). The extracts obtained were centrifuged (13,439 *g*, 10 min), and the supernatants were air-stream dried. The residues were redissolved in 1 mL dichloromethane:chloroform (1:1, v/v), centrifuged (13,439 *g*, 10 min), and the supernatants were air-stream dried. The residues were redissolved in 200 µL ethyl acetate and centrifuged (13,439 *g*, 10 min). The supernatants were transferred to GC–MS vials with glass inlet, and the ethyl acetate was air-stream dried. Residuals were resuspended in 50 µL N-methyl-N-(trimethylsilyl)-trifluoroacetamide (MSTFA; ABCR, Karlsruhe, Germany) and silyated for 30 min at 60 °C. Silyated samples were analyzed on a GC–MS at 70 °C for 3 min, 70–310 °C in 24 min [10 °C min^−1^], 310 °C for 5 min with a helium flow of 1 mL min^−1^, an injection volume of 1 µL and a split ratio of 1:10. Relative quantification of the individual compounds was achieved using the added internal standard 4-hydroxybiphenyl (response factor 1), which allowed a relative quantitative comparison of the levels of phytoalexin content in all samples. A set of co-injected hydrocarbons (even-numbered C14 to C32) was used to calculate the retention indices by linear extrapolation, as described in the literature (Busnena et al. 2021).

### Analysis of root morphology

For root morphological analyses, roots sampled were scanned at 720 dots per inch with 35 μm resolution using a flatbed scanner (EPSON perfection V700). Root traits were analyzed using the software WinRhizo 2019 (Regent Instruments, Canada). Root length was measured in 15 diameter classes divided in 100 µm intervals ranging from < 100 µm to > 1.4 mm. For statistical analysis, the four subsamples, and the three additional samples of entire root systems were considered. To check the quality of the measured root data, the root length of all samples included in the analysis was plotted against the respective root surface without any grouping (Fig. S1). All samples fitted to a linear relation indicating that the subsampling of root fractions (*n* = 4) was successful as the root-to-surface ratio was in line with that of entire root systems (*n* = 3). Thus, all 42 samples were included in the analysis of root morphology.

### Detection of inoculants

To check the colonization of the inoculated strains, serial dilutions of the detached cells from RA and RP were prepared 3, 16, and 28 dpi. Dilutions were plated and incubated for 48 h on media MB and MP for FZB42 and RU47, respectively. For the cultivation-independent quantification of total bacteria in RA and RP, a quantitative real-time PCR (qPCR) of the *16S rRNA* gene fragment was performed according to Suzuki et al. (2000) using RA and RP DNA extracts. The abundance of RU47 was quantified using newly developed primers (*aombb-F* and *aombb-R*) and a TaqMan probe (*aombb*-P) targeting an autotransporter outer membrane beta-barrel domain-containing protein encoding gene (*aombb*) (Eltlbany 2019). Details on primer and probe design and qPCR conditions are described in supplemental File S1 and supplemental Table S1. The relative abundance of RU47 was calculated based on the absolute abundances of *aombb* and *16S rRNA* gene copies. All reactions were performed on a CFX Connect real-time PCR cycler (Bio-Rad Laboratories Inc., Hercules, CA, USA).

### DNA extraction and amplicon sequencing

Microbial community DNA of 0.5 g RA and total RP (~ 0.1 g) samples was extracted using the FastPrep-24 bead-beating system and the FastDNA Spin Kit for Soil (MP Biomedicals, Eschwege, Germany) following the manufacturers’ instructions. DNA extracts were purified using the Geneclean Spin Kit (MP Biomedicals, Eschwege, Germany). For samples taken 28 dpi, the V3–V4 regions of bacterial and archaeal *16S rRNA* gene and the fungal ITS2 region were amplified from RA and RP DNA using primer pairs 341F/806R (Sundberg et al. 2013) and gITS7/ITS4 (White et al. 1990; Ihrmark et al. 2012). Samples were shipped to the sequencing service provider Novogene Co. (Cambridge, UK) where PCR amplification, library preparation, and sequencing were performed using Illumina MiSeq v2 PE250 according to the companies’ standard procedures.

For RE, frozen samples were homogenized using sterile metal beads (5 mm diameter) and a mixer mill MM400 (RETSCH GmbH, Haan, Germany) at 27 s^−1^ for 30 s. This step was repeated if the samples were not completely homogenized. DNA extraction was performed using the Invisorb Spin Plant Mini Kit (Invitek Molecular, Berlin, Germany), following the manufacturers’ instructions with the following modifications. (I) Samples were centrifuged at 11,000 *g* for 7 min before transferring them to the pre-filter to prevent filter clogging. (II) The elution step was performed in two steps with 50 µL elution buffer each instead of once with 100 µL. The *16S rRNA* gene fragment of the variable V3–V4 region was amplified using primers 335F/769R (Dorn-In et al. 2015), which were used to exclude amplification of *16S rRNA* genes derived from chloroplasts and mitochondria. The amplified DNA was sequenced on an Illumina MiSeq sequencer as described previously (Mahnkopp-Dirks et al. 2022) with the following modifications: PCR was done using NEBNext high-fidelity polymerase (New England Biolabs, Ipswich, USA) in a total volume of 25 µl (15 ng DNA template, 12.5 µl polymerase, 5 pmol of each primer) for 5 min at 98 °C; 30 cycles of 10 s at 98 °C, 30 s at 60 °C, 30 s at 72 °C; 5 min at 72 °C.

### Bioinformatic analysis

Datasets from *16S rRNA* gene and ITS amplicon sequencing were handled independently. For *16S rRNA* gene amplicons from RA and RP, paired-end reads were assigned to samples according to their unique barcodes and truncated by cutting off the barcode and primer sequences. Paired-ends were merged using FLASH (version 1.2.7) resulting in the overlapping splicing sequences, called raw tags. Quality filtering of the raw tags was performed using Qiime2 (Bolyen et al. 2019) to obtain high-quality clean tags. To detect chimera sequences, clean tags were compared with the Gold reference database (Edgar et al. 2011) using the UCHIME algorithm. Chimera sequences were removed, finally resulting in effective tags. For sequences derived from RE samples, the pipeline was modified as follows: Sequences were analyzed on the Galaxy web platform version 1.20 (Galaxy 2022). FASTQ files were trimmed with a minimum read length of 50 using Cutadapt (Martin 2011). Quality control was performed via FastQC. For subsequent data analysis, the DADA2 pipeline (Callahan et al. 2016) was used with the following trimming and filtering parameters: 20 bp were removed n-terminally, and reads were truncated at position 280 (forward) and 220 (reverse), with an expected error of 4 (forward) and 6 (reverse), respectively. Amplicon sequence variants (ASVs) were annotated using SILVA database version 132 (Quast et al. 2013). The primers for the *16S rRNA* gene were designed to bind to areas inside the bacterial domain but partial binding to the archaeal domain is also possible. We will refer to this subset of the microbiota as the bacterial community. For the analysis of ITS amplicons derived from RA and RP, PCR primers were trimmed of raw sequence reads. Read pairs in which any of the primers were not detected were discarded using cutadapt (version 2.3) (Martin 2011). Error-correction was performed for the trimmed sequence-reads, sequences were merged and ASVs were annotated using DADA2 (version 1.10.0) (Callahan et al. 2016) within Qiime2 (Bolyen et al. 2019). ASV annotation was done using UNITE database version 8.3 (Abarenkov et al. 2021). For all datasets, reads were excluded if classified as mitochondria, chloroplast, or plant tissue (mainly *Malus*) or if the phylum was missing. ASVs occurring in PCR no template controls were excluded as potential contaminants. For RA and RP, ASVs that were found uniquely in samples of treatments B or P and matched the genome sequence of FZB42 or RU47, respectively, were removed to depict the modulation of the microbiome without potential bias of ASVs affiliated to the inoculants. This was not done for RE, enabling to affirm if internal plant tissue was colonized by FBZ42 or RU47, a comparison between the sequences of the inoculants and the endophytes at ASV level was carried out in Bioedit (Hall 1999). The sequences were aligned using ClustalW Multiple alignment (Thompson et al. 1994) with 1000 bootstraps.

### Statistical analysis and data visualization

The analyses were performed using R version 4.2.1 (R Core Team 2022) in RStudio version 2022.02.1. (R Studio Team 2020). Pearson’s product-moment correlation was calculated to establish a correlation between CFU counts of RU47 and its relative abundance measured by qPCR. Analysis of the microbial community composition in RA, RP, and RE was performed on rarefied data following as recently recommended by Schloss (2024). Data were rarefied by randomly subsampling to the lowest number of reads for RA and RP samples: 40,118 for *16S rRNA* gene and 62,855 for ITS datasets and 18,788 for *16S rRNA* gene sequencing in RE using *phyloseq* (Love et al. 2014). Average α-diversity indices for species richness, diversity (Shannon), and evenness (Pielou) were calculated using *phyloseq* (Love et al. 2014) and *forcats* (Wickham 2021) packages. Data were checked for normal distribution by the Shapiro-Wilks test and considered normally distributed at an adjusted *p* > 0.05 (Benjamini-Hochberg correction). For normally distributed data, the significance of differences in α-diversities was tested by two-factorial analysis of variance (ANOVA) followed by Tukey’s HSD test. For non-normally distributed data, the Kruskal-Wallis test followed by Dunn’s test was applied using *vegan* (Oksanen et al. 2020) and *agricolae* (De Mendiburu and Yaseen 2020). Effects of microhabitat (RA and RP), soil, and treatment on the microbial communities were tested by permutational analysis of variance (PERMANOVA) based on Bray-Curtis dissimilarity using 10,000 permutations based on square root-transformed count data using *vegan* (Oksanen et al. 2020). Ordination of microbial community compositions was obtained by non-metric multidimensional scaling (NMDS) and Bray-Curtis dissimilarity based on square root-transformed count data using *vegan* (Oksanen et al. 2020). A negative binomial Wald test (Love et al. 2014) was used for differential abundance on rarefied reads to identify species with significant differences across RA, RP, and RE among the 20 most abundant ASVs in each microhabitat using DESeq2 v1.18.1 inside *phyloseq* (Love et al. 2014). Figures were generated using *ggplot2* (Wickham 2016), *ggpubr* (Kassambara 2020), *pheatmap* (Kolde 2019), and *RColorBrewer* (Neuwirth 2014) packages. For normally distributed parametric data of two variables, statistical differences were tested using paired *t*-test.

## Results

### Bacterial inoculants successfully colonized root-affected soil and rhizoplane

**Fig. 3:**
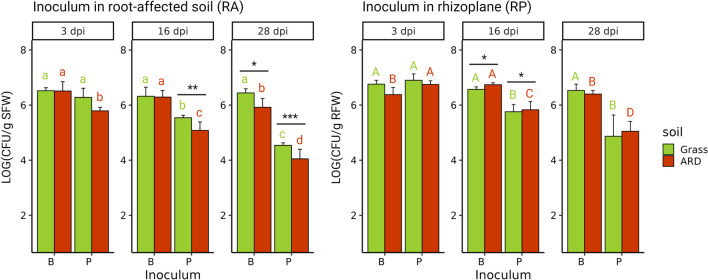
Colony-forming units (CFU) of the inoculated strains *Bacillus velezensis* FZB42 (B) and *Pseudomonas* sp. RU47 (P) 3, 16, and 28 days post inoculation (dpi) in root-affected soil and rhizoplane of apple M26 per gram soil fresh weight (SFW) or root fresh weight (RFW). Means of *n* = 7–12 with standard deviation are depicted. Different letters indicate significant differences within one soil (ARD or grass) 3, 16, and 28 dpi, according to Tukey’s HSD test. Minor letters mark differences in root-affected soil (RA), capital letters in rhizoplane (RP). Asterisks indicate significant differences between the two groups, comparing CFU counts of B or P in ARD and grass soils at one time point, according to paired *t*-test (*p < 0.05; **p < 0.01; ***p < 0.001)

The CFU counts determined on selective media for RA and RP 3, 16, and 28 dpi revealed that both strains established well in ARD and grass soils, indicating the successful colonization of the inoculants (ARD vs. grass: Fig. 3). Only for RU47 in RA, significantly lower CFU numbers were recorded in ARD soil 16 and 28 dpi. The CFU counts of FZB42 were stable over 4 weeks in RA ARD and grass soil, and only 28 dpi, the CFU counts in ARD soil were significantly lower compared to grass. In contrast, CFU counts of RU47 significantly decreased in RA over time with lower CFU counts 16 and 28 dpi in ARD soil compared to grass. Also, in RP, CFU counts of FZB42 remained stable over time at around 10^6^ CFU g^−1^ root fresh mass (RFW) for both, ARD and grass soils. Generally, CFU counts of RU47 were higher in RP than in RA but decreased significantly over time. Both inoculant strains successfully colonized RA and RP of apple plants M26 grown in both, ARD or grass soils for at least 4 weeks.

Bacterial *16S rRNA* gene abundance ranged from 5 × 10^7^ copies g^−1^ SFW to 2 × 10^8^ copies g^−1^ SFW in RA and 8 × 10^8^ copies g^−1^ RFW to 2 × 10^9^ copies g^−1^ RFW in RP. In RA and RP, absolute abundances of *16S rRNA* gene copies g^−1^ of SFW or RFM were not significantly different between treatments, soils, and time points. For the cultivation-independent detection of RU47, a qPCR was used to quantify *aombb* in RA and RP DNA extracts from 3, 16, and 28 dpi. In no sample of treatments C and B, amplification of specific *aombb* fragments was detected, confirming primer specificity. The qPCR data indicated a high competence of RU47 to colonize RP (Fig. S2). The strain established less well in RA. Already 3 dpi its relative abundance was at LOG(− 3.7) copies *aombb/16S rRNA* in ARD and LOG(− 3.6) copies *aombb/16S rRNA* in grass soil and further decreased significantly over time. At the same time points, no significant differences between grass and ARD soil were observed in RA. In RP, the abundance of RU47 was about 2 orders of magnitude higher 3 dpi compared to RA and the relative abundance of RU47 significantly decreased over time. There was a high positive correlation (*r* = 0.901, *p* < 0.001) between the results of the selective plating method using Log_10_-transformed CFU counts of RU47 (Fig. 3) and the copy numbers determined by qPCR of the *aombb* gene (Fig. S2).

### Microbial diversity in root-affected soil, rhizoplane, and root endosphere

Rarefaction curves (Fig. S3) showed that all samples reached a plateau with a sample size of > 20.000 reads per sample for *16S rRNA* gene and ITS amplicon sequencing of RA and RP and with > 5000 reads per sample for *16S rRNA* gene amplicon sequencing for RE. In all samples, the microbial diversity was covered sufficiently by the size of the sequence library. Calculating the α-diversity indices Shannon, richness and evenness for each microhabitat individually did not reveal differences for the bacterial or fungal diversity regarding the different treatments or soils (Fig. S4) except that significantly lower richness and Shannon indices were observed in the RP of apple plants grown in ARD soil compared to grass soil (C treatment). In the B and P treatments, α-diversity was restored.

**Fig. 4:**
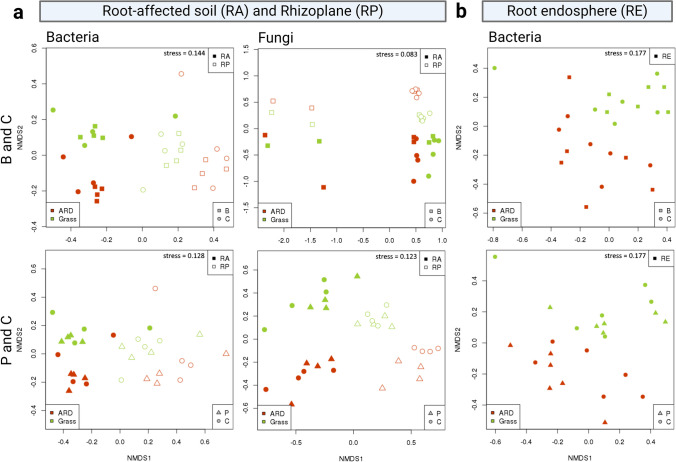
Non-metric-multidimensional scaling (NMDS) using a Bray-Curtis dissimilarity matrix on rarefied square root transformed count data of apple M26 treated with sterile H_2_O (C), *Bacillus velezensis* FZB42 (B), or *Pseudomonas* sp. RU47 (P) 28 dpi. **a** Bacterial and fungal β-diversity in root-affected soil (RA) and rhizoplane (RP). **b** Bacterial β-diversity in root endosphere (RE)

We analyzed the β-diversity for B and P in comparison to C, individually for each inoculant. The bacterial β-diversity was mainly shaped by the microhabitat (RA vs. RP), explaining 23% and 24% of the variance for B and P (Fig. 4A; Table 1). Also, the soil (ARD vs. grass) had a significant influence, explaining 6% of the variance for both B and P, while inoculation had no significant influence on bacterial β-diversity. Similar to the bacterial community, the fungal community composition was mainly shaped by microhabitat (RA vs. RP) followed by soil (ARD vs. grass) (Fig. 4A; Table 1). Both factors significantly influenced the fungal community composition irrespective of inoculation. However, a significant effect of the inoculum on fungal β-diversity, explaining 10% and 4% of the variance was observed for treatment B and P, respectively (Fig. 4A; Table 1). In RE, no evident clustering of samples separating treatments B and P from C was observed using NMDS-plotting (Fig. 4B), but the RE of plants grown in grass or ARD soils were clearly distinguished. This is supported by the results of PERMANOVA, showing highly significant differences between ARD and grass soils (Table 1).

**Table 1:** Permutational analysis on variance (PERMANOVA) on rarefied square root transformed count data assessing significance of microhabitat, soil (ARD or grass), and treatment (inoculation with B or P) on the bacterial and fungal β-diversity in root-affected soil (RA) and rhizoplane (RP) as well as of soil (ARD or grass) and treatment (inoculation with B or P) for the bacterial β-diversity in the root endosphere (RE)

		B and C		P and C	
		*R*^2^	*p*-value	*R*^2^	*p*-value
RA and RP					
Bacteria	Microhabitat (RA vs. RP)	0.23	< 0.001	0.24	< 0.001
	Soil (grass vs. ARD)	0.06	0.007	0.06	0.012
	Inoculum	0.04	0.09	0.04	0.07
Fungi	Microhabitat	0.15	< 0.001	0.20	< 0.001
	Soil	0.08	0.003	0.13	< 0.001
	Inoculum	0.10	< 0.001	0.04	0.041
RE					
Bacteria	Soil	0.24	< 0.001	0.21	< 0.001
	Inoculum	0.04	0.36	0.04	0.38

### Effect of soil and inoculation on dominant taxa in root-affected soil, rhizoplane, and root endosphere

**Fig. 5:**
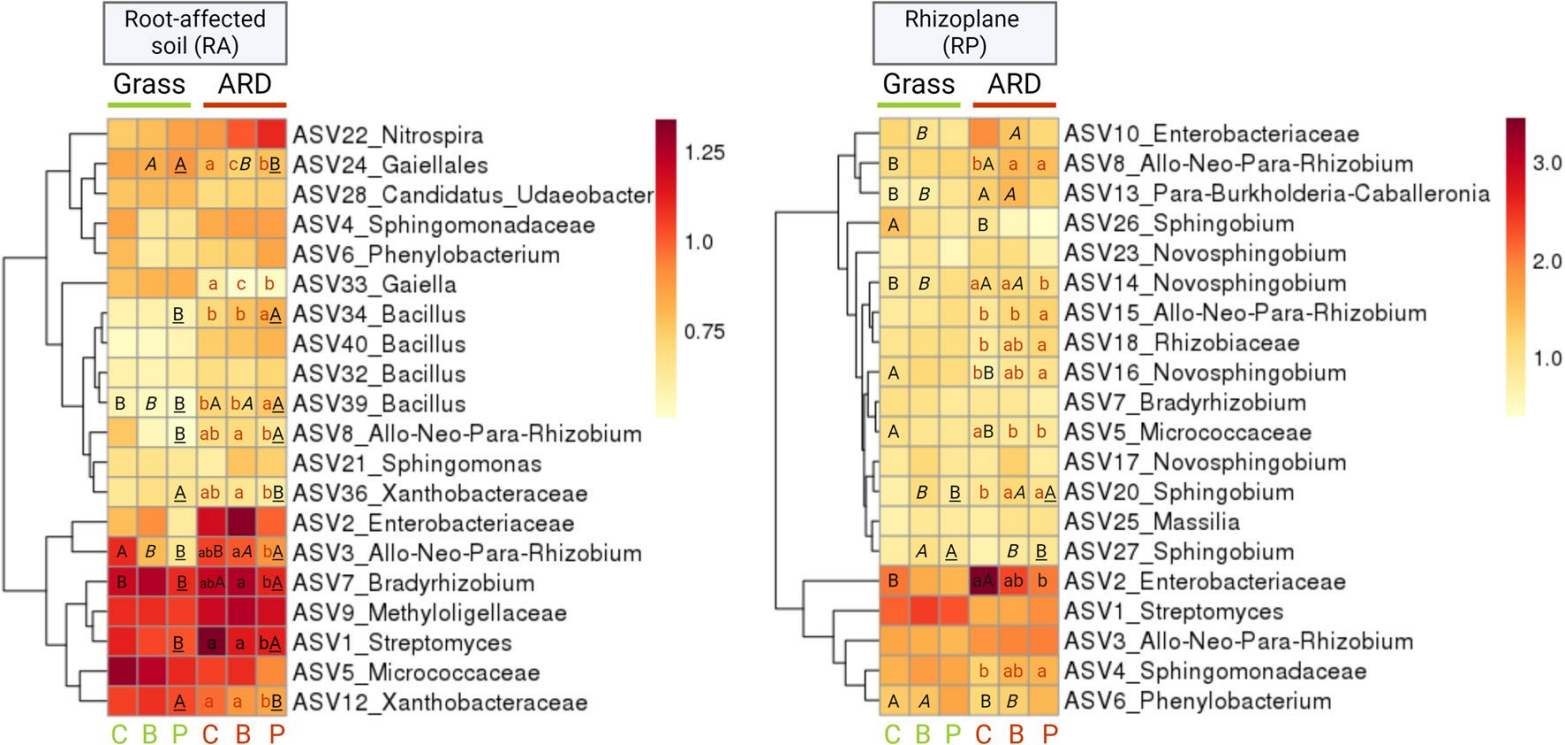
The 20 most abundant bacterial ASVs in root-affected (RA)-soil (left) and rhizoplane (RP) (right). Average values of rarefied square root transformed relative abundances of *n* = 4 are depicted. C: plants treated with sterile H_2_O, B: plants treated with *Bacillus velezensis* FZB42, P: plants treated with *Pseudomonas* sp. RU47. Different minor letters indicate significant differences between treatments of the same soil: C, B, and P in grass (green) or ARD (red). Capital letters indicate significant differences between grass and ARD soils within the three treatments: ARD_C vs. Grass_C (latin); ARD_B vs. Grass_B (italic); ARD_P vs. Grass_P (underlined). The significance of differences was tested based on pairwise comparison using generalized linear models in DeSeq2

The most pronounced differences in the relative abundance of the dominant bacterial taxa were observed between grass and ARD soils, irrespective of the different treatments in RA and RP (Fig. 5; Table S2). In RA, *Bacillus* (ASV39) and *Bradyrhizobium* (ASV7) were higher in relative abundance in ARD_C compared to Grass_C, while *Allorhizobium-Neorhizobium-Pararhizobium-Rhizobium* (ASV3) was significantly lower in ARD_C than in Grass_C. In RA of ARD soil, the relative abundance of *Gaiellales* (ASV24) and *Gaiella* (ASV33) was significantly higher in ARD_C compared to ARD_B and ARD_P. Two ASVs affiliated to *Bacillus* (ASV34 and ASV39) were significantly lower in relative abundance in ARD_C and ARD_B compared to ARD_P. Contrary, the relative abundance of *Streptomyces* (ASV1) was significantly higher in ARD_C and ARD_B compared to ARD_P.

In RP, bacterial ASVs of *Streptomyces* (ASV1), *Novosphingobium*, or taxa belonging to the families *Enterobacteriaceae* and *Sphingomonadaceae* were overall highly abundant in both soils and all treatments (Fig. 5; Table S2). The relative abundances of *Burkholderia-Caballeronia-Paraburkholderia* (ASV13) and *Enterobacteriaceae* (ASV2) were significantly higher in RP of ARD_C compared to Grass_C. In contrast, the relative abundance of *Sphingobium* (ASV26) and *Phenylobacterium* (ASV6) was significantly lower in ARD_C than in Grass_C. Four of the 20 most abundant bacterial ASVs in RP affiliated to *Novosphingobium* (ASV14, ASV16, ASV17, and ASV23) were differently abundant either in ARD or grass soil. A taxon from the family *Enterobacteriaceae* (ASV2) was higher in relative abundance in ARD_C (11.8%) compared to ARD_B (5.9%) and ARD_P (4.2%), with ARD_P being significantly lower. The same trend was observed for the relative abundance of *Enterobacteriaceae* (ASV10) which was highest in ARD_C (3.69%), followed by ARD_B (1.82%) or ARD_P (1.32%). Interestingly, in both, RA and RP, the different treatments significantly affected the relative abundance of dominant ASVs exclusively in ARD soil while no significant differences between treatments were observed in grass soils.

**Fig. 6:**
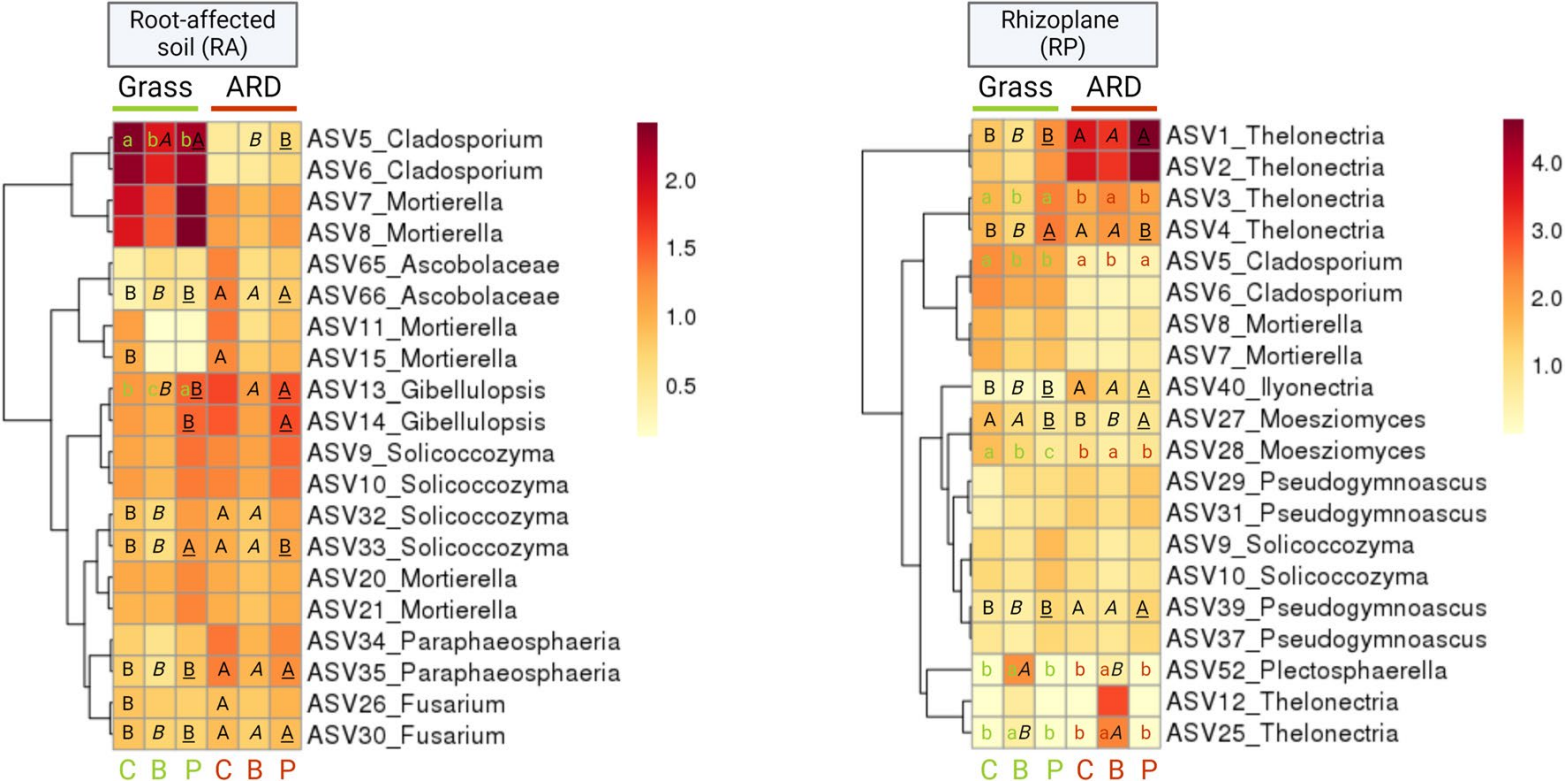
The 20 most abundant fungal ASVs in root-affected (RA)-soil (left) and rhizoplane (RP) (right). Average values of rarefied square root transformed relative abundance of *n* = 4 are depicted. C: plants treated with sterile H_2_O, B: plants treated with *Bacillus velezensis* FZB42, P: plants treated with *Pseudomonas* sp. RU47. Different minor letters indicate significant differences between treatments of the same soil: C, B, and P in grass (green) or ARD (red). Capital letters indicate significant differences between grass and ARD soil within the three treatments: ARD_C vs. Grass_C (latin); ARD_B vs. Grass_B (italic); ARD_P vs. Grass_P (underlined). The significance of differences was tested based on pairwise comparison using generalized linear models in DeSeq2

As described for bacteria, the most pronounced differences among the most abundant fungal ASVs were observed between ARD and grass soil in both microhabitats, RA and RP (Fig. 6; Table S3). In RA, pairwise comparisons of each treatment revealed that *Mortierella* (ASV15) was significantly higher only in ARD_C compared to Grass_C, but not after treatments B or P. Overall, a clear trend that indicated lower relative abundances of ASVs identified as *Mortierella* (ASV5, ASV6, ASV7, and ASV8) in ARD_C compared to Grass_C was observed. In RA from grass, only the relative abundance of *Cladosporium* (ASV5) was significantly lower after treatment B and P compared to treatment C. The present dataset revealed that in RP, *Thelonectria* (ASV1, ASV2, ASV3, and ASV4) and *Ilyonectria* (ASV40) were dominant in all treatments with ASV1, ASV4, and ASV40 being significantly higher in relative abundance in RP from ARD soil compared to grass for all treatments except ASV4 in treatment P. In the RP of grass soil, the relative abundance of *Cladosporium* (ASV5) and *Moesziomyces* (ASV28) was significantly higher in treatment C than in treatments B or P.

**Fig. 7:**
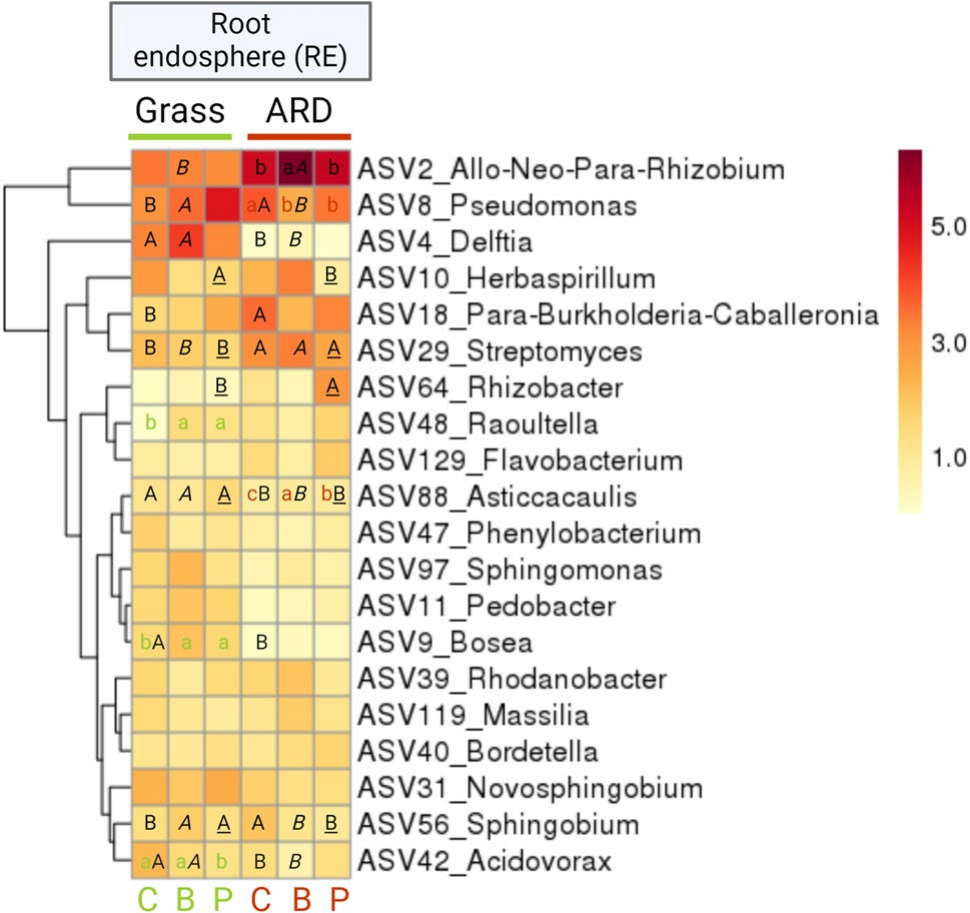
The 20 most abundant bacterial ASVs in root endosphere (RE) of apple M26. Average values of rarefied square root transformed relative abundance of *n* = 6 are depicted. C: plants treated with sterile H_2_O, B: plants treated with *Bacillus velezensis* FZB42, P: plants treated with *Pseudomonas* sp. RU47. Different minor letters indicate significant differences between treatments of the same soil: C, B, and P in grass (green) or ARD (red). Capital letters indicate significant differences between grass and ARD soils within the three treatments: ARD_C vs. Grass_C (latin); ARD_B vs. Grass_B (italic); ARD_P vs. Grass_P (underlined). Significance of differences was tested based on pairwise comparison using generalized linear models in DeSeq2

In RE, differences in relative abundances among the most abundant ASVs were observed mainly between ARD and grass soil (Fig. 7; Table S4). The relative abundance of *Pseudomonas* (ASV8) was significantly higher in ARD_C compared to Grass_C as well as compared to ARD_B and ARD_P. The relative abundance of *Burkholderia-Caballeronia-Paraburkholderia* (ASV18) was significantly higher in ARD_C (12.6%) than in Grass_C (2.3%). The relative abundance of *Streptomyces* (ASV29) was significantly increased in RE of ARD soils compared to grass in all treatments. In RE of all ARD soils, *Bosea* (ASV9) was low in relative abundance in all treatments (< 0.1%). It was mainly present in grass soil and significantly lower in relative abundance in Grass_C (2.4%) compared to Grass_B (4.1%) and Grass_P (2.5%). *Delftia* (ASV4) was almost exclusively present in the RE of grass soil. It accounted for 10.4%, 12.7%, and 10.0% of the relative abundance in RE of Grass_C, Grass_B and Grass_P, but only for 0.01%, 0.16%, and 0% in ARD_C, ARD_B and ARD_P, respectively. In RE, the relative abundance of two bacterial ASVs was significantly different due to inoculation with B or P in RE from either ARD or grass soil. In comparison, in RA and RP a higher number of the dominant bacterial ASVs was significantly different due to inoculation, exclusively in ARD but not in grass soil.

The comparison of ASVs affiliated with the genus *Bacillus* with the genome sequence of FZB42 revealed that the sequence of ASV1040 overlapped 100% with the sequence of inoculant FZB42. Based on amplicon sequencing analysis, ASV1040 was annotated as *Bacillus velezensis* and occurred exclusively in RE samples of ARD_B and Grass_B with a relative abundance between 0.052 and 0.228%. For RU47, ASV50 overlapped 100% with at least one of the multiple *16S rRNA* gene copies of the RU47 genome. It occurred almost exclusively in samples of treatment P, with one exception where it occurred in relatively low abundance in one sample of Grass_C. Based on the results of amplicon sequencing, ASV50 was annotated as *Pseudomonas baetica* and occurred with a relative abundance between 0.064 and 8.61%. The data indicate that the inoculants FZB42 and RU47 were detected in the RE. Altogether, inoculation with FZB42 or RU47 did not lead to a significantly increased relative abundance of the genera *Bacillus* or *Pseudomonas* in RE of treatments B or P.

### Phytoalexins

**Fig. 8:**
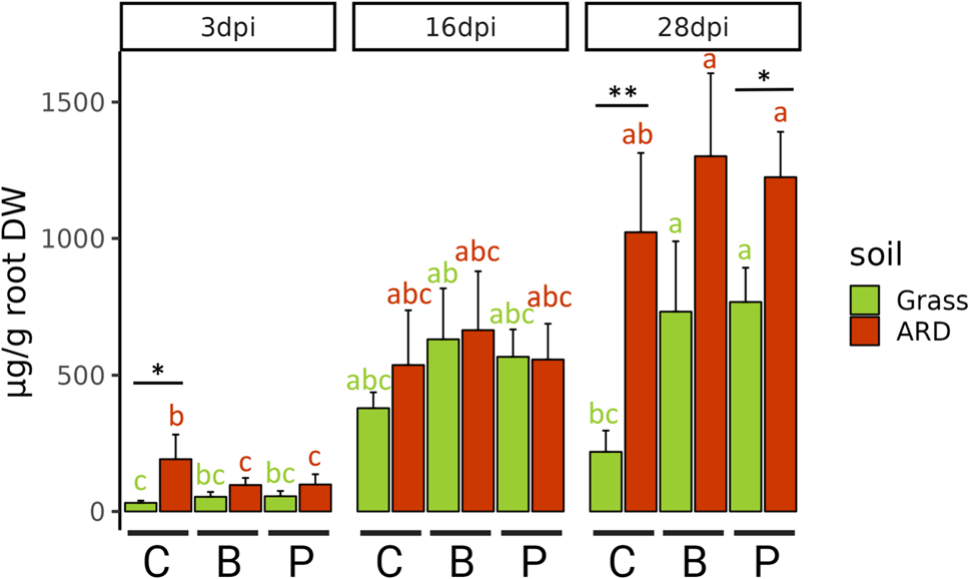
Total phytoalexins per gram root dry weight (RDW) of apple M26 3, 16, and 28 days post inoculation (dpi) treated with sterile H_2_O (C), *Bacillus velezensis* FZB42 (B), or *Pseudomonas* sp. RU47 (P) grown in ARD (red) or grass (green) soils. Means of *n* = 4 with standard deviation are depicted. Asterisks indicate significant differences between two groups according to paired *t*-test (*p* < 0.05*; *p* < 0.01**)

In general, the total PA content in roots of M26 increased over 4 weeks, resulting in significantly higher PA contents 28 dpi compared to 3 dpi (Fig. 8). At all time points, the content of PAs in the roots was the lowest in Grass_C. Three dpi, the total PA content in roots of ARD_C (191.7 µg g^−1^ root dry weight (RDW)) was significantly higher than that in roots of Grass_C (31.6 µg g^−1^ RDW; *p* < 0.05). In contrast, no significant differences existed in total PA content between ARD_B and Grass_B, ARD_P and Grass_P. When comparing the treatments 3 dpi, the total PA content in ARD_C was significantly higher than those of ARD_B and ARD_P. In contrast, no significant effect of the inoculation treatments was observed in grass soil. Sixteen dpi, PA contents in roots increased in all variants with no significant differences between treatments and soils. Twenty-eight dpi, PA contents in roots of ARD_C (1022.7 µg g^−1^ RDW) and ARD_P (1224.4 µg g^−1^ RDW) were significantly higher than those of Grass_C (218.8 µg g^−1^ RDW) and Grass_P (768.4 µg g^−1^ RDW). Interestingly, the total PA contents in Grass_B (733.0 µg g^−1^ RDW; *p* < 0.05) and Grass_P (768.1 µg g^−1^ RDW; *p* < 0.05) were significantly higher than that of Grass_C (218.8 µg g^−1^ RDW). Peak PA concentrations were measured in ARD soils at the latest time point in ARD_B (1301.3 µg g^−1^ RDW), ARD_P (1224.4 µg g^−1^ RDW). No significant differences were detected among the treatments in ARD soil with PA contents in ARD_C (1022.7 µg g^−1^ RDW) being slightly lower than in ARD_B and ARD_P. These results indicate that inoculation with B or P to apple plants grown in grass soil significantly induced the production of PAs in the roots at the late time point (28 dpi). The inoculation with B or P to apple plants grown in ARD soil did not significantly, but slightly increase PA production.

The dominant PA was 2-hydroxy-4-methoxydibenzofuran in all variants (Fig. S5). Noraucuparin and 3-hydroxy-5-methoxybiphenyl ranked second and third. Over time, the concentrations of hydroxyeriobofuran isomer 2 and noreriobofuran in roots increased. Three dpi, the PAs at the end of their biosynthetic pathway, like noreriobofuan and eriobofuran, were absent in all variants. Still, 16 dpi and 28 dpi, they appeared in all variants except for Grass_C with increased contents 28 dpi compared to 16 dpi.

### Root morphology

Root length was not significantly different between treatments or when comparing root systems grown in ARD or grass soil (Fig. S6). However, some trends were observed: longest root systems were present in Grass_C. The length of inoculated root systems was reduced to 69% in Grass_B and 58% in Grass_P compared to Grass_C. This reduction was not observed for ARD soils: root systems grown in ARD soils were smaller and relative root length was 40–50% lower in ARD soils compared to Grass_C with no effect of inoculation (Fig. S6). A comparison of root diameters measured in 100 µm steps indicated that growth in ARD soil led to the formation of significantly less fine roots and higher portions of thicker roots compared to Grass_C, which was observed for all treatments (Fig. S7). Inoculation with both B and P resulted in significant shifts in root morphology towards less fine roots and higher portions of thicker roots in inoculated root systems, exclusively in grass, but not in ARD soil.

## Discussion

The current study demonstrated the competence of *Pseudomonas* sp. RU47 and *Bacillus velezensis* FZB42 to colonize the rhizoplane and root-affected soil of apple plants grown in ARD or grass soil. This phenomenon was observed through cultivation-dependent methods for FZB42 and RU47 and cultivation-independent methods for RU47. Twenty-eight dpi, amplicon sequencing data indicated that the inoculants colonized also the root endosphere. Inoculation resulted in a soil- and inoculant strain-dependent plant response. Plants responded strongly to the inoculants by increasing phytoalexins in roots mainly in grass soil. Soil, rhizoplane, and root endosphere microbiome modulation by the inoculant strains investigated by amplicon sequence analysis showed that the microhabitat (RA vs. RP) and the soil (ARD vs. Grass) shaped the microbiome more strongly than the inoculants.

### Application of bacterial inoculants in apple cultivation

To our knowledge, this study is the first one to describe an inoculation procedure for the two bacterial strains on apple plants, which can be easily transferred from greenhouse to field scale. Root dipping can be easily implemented in apple tree planting in tree nurseries and orchards. Usually, roots of the apple plants are stored in a water bath the night before planting which can be supplemented with the bacterial inoculants, causing no additional work for farmers. For repeated inoculation during the growing period, the inoculum suspension could be applied by drenching around the trees with little effort. Successful drenching was demonstrated by Utkhede et al. (2001) with *Bacillus subtilis* EBW4 upon planting apple trees in an orchard in British Columbia, which led to significantly increased fruit yield, reduced disease severity and improved trunk radial growth compared to untreated apple plants. Recently, two other bacterial strains of the genus *Bacillus* were isolated from healthy apple roots from ARD-affected soils in China (Duan et al. 2021, 2022a). Both strains were investigated for their biological control ability with a focus on the potential to reduce the growth and germination of different *Fusarium* species that are assumed to add to the ARD disease complex in Chinese soils (Duan et al. 2022a, 2022b). Both studies mainly focused on *in vitro* experiments and did not assess the inoculants rhizosphere competence or potential to modulate the microbiome. Several studies claimed that *Fusarium* was one of the primary causal pathogens of ARD in China (Wang et al. 2018; Jiang et al. 2022). However, there are no studies so far providing clear evidence for the contribution of *Fusarium* to ARD as discussed by Somera and Mazzola (2022). Our study revealed two ASVs assigned to *Fusarium* to occur in significantly higher relative abundance in ARD than grass soil. Regarding the relative abundances however, these differences were small (~ 0.1%). Moreover, the increased relative abundance of a certain taxon, here *Fusarium*, does not provide evidence for the contribution of this taxon to ARD, as the data are based on amplicon sequencing only. Furthermore, previous microscopic studies employed on the same ARD soil did not find evidence for an important role of *Fusarium* in ARD soils from three sites in Germany (Grunewaldt-Stöcker et al. 2021). While the studies mentioned above investigated inoculation effects on plant growth, fruit yield or reduction of potential pathogens, our study is the first to examine the modulation of the microbial community composition of apples across microhabitats, phytoalexin content and root morphology triggered by two bacterial inoculants.

### Colonization competence and quantification techniques of the inoculant strains

The present study provides first evidence that both, FZB42 and RU47, are rhizosphere competent on apple rootstock M26 for at least 4 weeks. The use of rifampicin-resistant mutants allowed a specific cultivation-dependent quantification of the inoculants. Compared to amplicon sequencing of the *16S rRNA* gene, CFU counting is highly sensitive and provides actual numbers of CFU instead of only relative abundances. The CFU counting method is fast, cost-effective, and easily applicable in every wet lab. In addition to selective plating, a TaqMan-based qPCR-system was developed to quantify RU47. In contrast to the spore-former FZB42, the cultivability of RU47 can be affected by environmental stress and therefore a DNA-based quantification for this strain was also used. We propose that the newly developed qPCR system allows specific detection of the inoculant strain in rhizoplane and soil of RU47-inoculated apple plants. In this study, the results of CFU counting and quantification of the *aombb* gene of RU47 were highly positively correlated, indicating that the newly developed qPCR assay provides reliable and accurate results for the cultivation-independent quantification of RU47. Combining selective plating and qPCR complements the benefits of both methods. While CFU counting is more sensitive at a lower detection limit than qPCR, the latter allows the detection of cells in a non-cultivable state.

Additionally, amplicon sequencing data indicated that both, RU47 and FZB42, colonized the root endosphere. Generally, roots are considered the most common entry point for the colonization of the root interior of plants, including apple (Frank et al. 2017). Previously, Chen et al. (2013) were able to detect the inoculants *Burkholderia* sp. and *Pseudomonas thivervalensis* in the root interior of rape 60 dpi. Lacava et al. (2007) showed that citrus tree roots (*Citrus sinensis*) inoculated with *Klebsiella pneumoniae* Kp342 colonized the root interior. The present study revealed two ASVs exclusively in samples of treatments B (ASV1024) and P (ASV50) which were identical with genome sequences of the inoculant strains FZB42 and RU47, respectively. This indicates the potential colonization of the root endosphere by the inoculant strains. However, due to the short sequences obtained by amplicon sequencing, the approach does not allow an identification of the inoculants on species or strain level leaving some uncertainty regarding their identification. Nonetheless, considering the ability of FZB42 and RU47 to colonize soil and rhizoplane, we conclude that the inoculant strains are likely to successfully colonize the root endosphere.

### Differences in microbial community composition in ARD and grass soil

The microbial community composition of ARD and grass soil from the same experimental site used in this study was described previously for different microhabitats (Balbín-Suárez et al. 2020, 2021; Mahnkopp-Dirks et al. 2021). As described before (Balbín-Suárez et al. 2020), the composition of bacteria was mainly driven by the microhabitat and significantly differed between rhizoplane and root-affected soil. These results support the expectation that the microhabitat marks the main differences in microbial community composition (Reinhold-Hurek et al. 2015; van der Heijden and Schlaeppi 2015). Balbín-Suárez et al. (2020, 2021) identified several taxa to be higher in abundance in either ARD or grass soil and identified taxa that uniquely responded to the respective soil. In ARD soil or rhizoplane, they identified *Burkholderia*, *Variovorax*, *Streptomyces*, and *Nectria* in increased relative abundance. The present study showed ASVs affiliated with *Burkholderia-Caballeronia-Paraburkholderia*, *Thelonectria*, and *Ilyonectria* to be of significantly higher relative abundance in the rhizoplane of plants grown in ARD soils. The potential contribution of *Nectriaceae* to the ARD disease complex has recently been studied intensively (Popp et al. 2019, 2020) and is further supported by the notably high occurrence of four ASVs (fungal ASV1-ASV4 in rhizoplane) belonging to the genus *Thelonectria* and ASV40 belonging to the genus *Ilyonectria* in the present dataset.

Our study found ASVs affiliated to the order *Enterobacteriaceae* highly abundant in soil and rhizoplane of apple plants grown in ARD soil. *Enterobacteriaceae* were not observed before in rhizoplane and soil originating from the same site (Balbín-Suárez et al. 2020, 2021), but one ASV identified as *Enterobacteriaceae* was found in the root endosphere of apple grown in ARD soil from the same site that negatively correlated with shoot growth and shoot fresh mass (Mahnkopp-Dirks et al. 2021). In the rhizosphere and the root endosphere of apple trees affected by bitter rot and leaf spot disease in Brazil, Dos Passos et al. (2014) found cultivable *Enterobacter* to be the most abundant genus. Members of the family *Enterobacteriaceae* can indirectly influence a plant’s defense response by supporting plant pathogens and have the potential to decompose plant tissue (Berg et al. 2017). In the treatments with the bioinoculants a lower relative abundances of *Enterobacteriaceae* than non-inoculated controls which could be an indicator for successful modulation of bacteria that accumulate in ARD-affected soils. Certain ASVs of *Rhizobium* and *Streptomyces* were previously reported to occur in increased relative abundances in apple roots grown in ARD soil (Mahnkopp-Dirks et al. 2021). In the present study, *Streptomyces* was one of the most abundant taxa in the root endosphere, with ASV29 being significantly higher in relative abundance in roots from ARD than in grass soil. Our study revealed a strikingly lower relative abundance of *Delftia* of almost 0% in ARD soils, which was not observed before. Endophytic strains of the genus *Delftia* are commonly associated with plant beneficial traits (Han et al. 2005; Woźniak et al. 2018, 2019; Da Silveira et al. 2019; Chen et al. 2020; Islam et al. 2023). We assume that a reduction of endophytic *Delftia* in ARD-affected apple roots might contribute to the plant growth depression. Similarly, also the relative abundance of *Bosea* was remarkably low with almost 0% in ARD soils, but was found in relative abundances of 2.5% or higher in grass soils. The genus *Bosea* belongs to the novel family *Bosaceae* that recently emanated from the family *Bradyrhizobiaceae* (Hördt et al. 2020)*.* Most members of *Bosaceae* were isolated from legume nodules pointing towards a close association between *Bosea* and legumes. Also, the co-existence of endophytic *Bosea spatocytisi* sp. nov. with rhizobia in root nodules was observed (Pulido-Suárez et al. 2022). The composite mix of plants used as grass cover in our study site included *Trifolium*, which might explain why *Bosea* was observed exclusively in samples from grass, but not ARD soils.

In rhizoplane of grass soil, e.g., *Sphingobium* and *Novosphingobium* occurred in significantly higher relative abundance than in rhizoplane of ARD soil. Several members of the genera *Sphingobium* and *Novosphingobium* are well-known environmental strains involved in bioremediation and biodegradation (Yang et al. 2014; Boss et al. 2022). Also, various isolates of the family *Sphingomonadaceae* were identified as PGPR with the potential to produce phytobeneficial compounds or to increase root and shoot length when applied as inoculant (Yang et al. 2014; Krishnan et al. 2017). The significant higher relative abundance of *Sphingobium* and *Novosphingobium* in grass soil could have promoted the root growth observed in grass soils. To validate this hypothesis, isolation, *in vitro* testing, and inoculation experiments using *Sphingomonadaceae* isolates would be needed.

### Microbiome modulation of the microbial community composition

Over the past years, biocontrol, inoculation of beneficial microbes, and measures to increase microbial diversity and activity have gained increasing attention as eco-friendly management options in agriculture (Berg et al. 2017; Tosi et al. 2020; Müller and Behrendt 2021). Recently, microbiome modulation was introduced as an effective mode of action for microbial inoculants (Berg et al. 2021). Six different types of microbiome modulation were formulated including the restoration of a dysbiosis, the targeted shift towards potentially beneficial taxa, or the depletion of potential pathogens (Berg et al. 2021). These authors summarized that the degree of microbiome modulation highly depends on the sampling time and mode of inoculant application and that shifts are usually only evident shortly after inoculation. This might explain why in our study no or only little effects of the inoculants on the bacterial ß-diversity were observed 28 dpi. However, fungal ß-diversity was significantly affected by both inoculants likely due to the antifungal activities of both inoculant strains. For instance, the potential to suppress the fungal plant pathogen *Rhizoctonia solani* was previously demonstrated for both, FZB42 and RU47 (Chowdhury et al. 2015; Schreiter et al. 2018). The significant shifts in fungal β-diversity as well as changes in relative abundances of the dominant fungal taxa indicate that FZB42 and RU47 modulated the fungal microbiome in both, ARD and grass soil. These results give perspective for future experiments in which the potential of the inoculants to suppress ARD-associated microorganisms like members of *Ilyonectria*, *Thelonectria*, or *Pythium* should be evaluated not only by amplicon sequencing-based approaches, but by *in vitro* studies following Koch’s postulates and molecular quantification tools such as qPCR assays targeting taxa that potentially contribute to ARD. Interestingly, regarding the dominant bacteria, only in root-affected soil and rhizoplane of ARD soil, many taxa that were detected changed significantly in relative abundance due to the inoculation while no significant changes in relative abundance were observed for any of the 20 most abundant taxa in both microhabitats from grass soil. We assume that due to the imbalanced microbiome of the ARD-affected soil and rhizoplane, the microbiome was less stable and could be modulated more easily.

### Plant stress response and plant growth are affected by inoculants

The production of phytoalexins is a common plant defense strategy to combat pathogen invasion. Apple and other rosaceous plants, in particular, form biphenyls and dibenzofurans to inhibit microbial growth and cell propagation in a local environment around the plant (Chizzali and Beerhues 2012; Busnena et al. 2023). Balbín-Suárez et al. (2021) showed that biphenyl and dibenzofuran phytoalexins predominantly and significantly accumulate in diseased roots of apple grown in ARD soils. This result was confirmed in the present study as significantly higher PA contents were observed in the roots of apple plants grown in ARD soil compared to grass soil. However, our results showed that phytoalexins were also induced by the inoculation of FZB42 or RU47 in the roots of apple plants especially in grass soil. Hence, the overall production of biphenyl and dibenzofuran phytoalexins seems not a specific response of apple roots to ARD stress but indicating an unbalanced microbiome. Phytoalexin production upon exposure to microbes is a general defense response of plants, which is also true for apple (Busnena et al. 2023). In 2017, Weiß et al. reported for the first time that apple roots form biphenyl and dibenzofuran phytoalexins upon microbial stress, here ARD soil. However, production of these phytoalexins after application of microbial inoculants, such as the two bacteria used in this study, has not been reported before. In the few years from 2017 up to now, to our knowledge, there was no report about the formation of the phytoalexins in apple roots in response to inoculants. We assume that the inoculation of high cell or spore numbers disturbed the previous equilibrium of the native soil microbial communities in grass soil, which might have led to the induction of the apple roots’ stress response. The increase in phytoalexin content in the FZB42 and RU47 treatments was weaker in the roots of plants grown in dysbiotic ARD soil. Further detailed research investigating the response of apple plants to microbial inoculants applied in different cell or spore densities is needed to answer this question.

Analyzing root length in the different soils and treatments did not reveal significant differences. Most likely, this can be explained by high plant-to-plant variations, impeding the detection of significant differences. However, we observed some trends that might indicate a link to phytoalexin contents. Twenty-eight dpi, roots that were inoculated had significantly higher phytoalexin contents compared to the respective non-inoculated controls in grass soil. We assume that the high phytoalexin content in roots of inoculated plants might have affected root growth. However, as the results regarding the root length were not significant, these observations need to be considered with care. Moreover, inoculation led to significantly higher proportions of thicker roots compared to the control only in grass soil which might have been caused by changes in the microbial community composition and activity. High ethylene concentrations are known to increase cortex width and hence root diameters (Gebauer et al. 2021). Here, it can only be speculated whether the differences in root diameter classes observed in the present experiment between grass soil and ARD soil are related to the documented differences in microbiome composition and their potential functional differences in hormone production.

In summary, we explored the potential of two microbial inoculants, *B. velezensis* FZB42 and *Pseudomonas* sp. RU47, on apple, to mitigate apple replant disease. We used inoculation techniques that enabled the inoculants to colonize different root-associated microhabitats of apple, which at the same time, can be easily implemented in current horticultural practices. As a first step, we showed that both inoculant strains had the potential to establish across microhabitats, to modulate the microbiome and to induce shifts in the relative abundance of dominant bacterial and fungal ASVs. The effects of microhabitat and replanting history of the soils on the bacterial community were stronger than the inoculation effect. To our surprise, the inoculation effect of FZB42 on the fungal community composition was stronger than the effect of replanting history likely due to the strong antifungal capacity of FZB42, potentially enabling the suppression of ARD-associated fungi. The inoculants decreased the relative abundance of ARD-related *Enterobacteriaceae*. Unexpectedly, inoculation increased phytoalexin content in roots of apple plants grown in grass soil. Whether increased phytoalexin contents can be linked to changes in root morphology that were observed needs additional investigation. To further unravel the interplay between inoculants and plants, and in particular their potential to reduce ARD, further field experiments and additional methods such as the measurement of volatile organic compounds or functional microbiome analysis, are needed. It will also be essential to investigate long-term effects of the inoculants in field trials.

## Supplementary information

Below is the link to the electronic supplementary material.

Supplementary file1 (PDF 1.08 MB)

## Data Availability

Raw data of *16S rRNA* gene and ITS amplicon sequencing including metadata were deposited at the NCBI under project number BioProject PRJNA1043463: https://www.ncbi.nlm.nih.gov/sra/PRJNA1043463.
