# Supplementary material for: Rhizosphere competent inoculants modulate the apple root–associated microbiome and plant phytoalexins
Source: Appl Microbiol Biotechnol. 2024 May 27;108(1):344. doi: 10.1007/s00253-024-13181-8 (PMC11129989; doi:10.1007/s00253-024-13181-8)
Supplement: Supplementary file 1 — Supplementary file1 (PDF 1.08 MB) [file 253_2024_13181_MOESM1_ESM.pdf]

Supplementary Materials

Applied Microbiology and Biotechnology

Rhizosphere competent inoculants modulate the apple root-associated microbiome and plant phytoalexins

Kristin Hauschild[1], Nils Orth[2], Benye Liu[3], Adriana Giongo[1], Silvia Gschwendtner[4], Ludger Beerhues[3], Michael Schloter[4], Doris Vetterlein[5], Traud Winkelmann[2] and Kornelia Smalla[1]*

[1] Institute for Epidemiology and Pathogen Diagnostics, Julius Kühn Institute, Federal Research Centre for Cultivated Plants, Braunschweig, Germany

[2] Institute of Horticultural Production Systems, Leibniz University Hannover, Hannover, Germany

[3] Institute of Pharmaceutical Biology, Technische Universität Braunschweig, Braunschweig, Germany

[4] Research Unit Comparative Microbiome Analysis, Helmholtz Centre Munich, Munich, Germany

[5] Department of Soil System Science, Helmholtz Centre for Environmental Research, Halle/Saale, Germany

*** Correspondence:**
Prof. Dr. Kornelia Smalla:
E-Mail: kornelia.smalla@julius-kuehn.de
Tel. (+49) 3946 47 6160
Fax: (+49) 3946 47 6106

Supplemental file S1: Development of quantitative real-time PCR assay for the quantification of strain *Pseudomonas* sp. RU47

A new set of primers and specific TaqMan probe for the quantification of *Pseudomonas* sp. RU47 were established and were previously described in the dissertation of Eltlbany (2019). Based on the RU47 genome (Kuzmanović et al. 2018) a primer set and a TaqMan probe targeting an autotransporter outer membrane beta-barrel domain-containing protein encoding gene (*aombb*) were designed and tested *in silico* as well as with a collection of *Pseudomonas* strains for specific amplification (Table S1). The *aombb* gene was extracted from the RU47 genome sequence, giving no significant hits based on BLASTN analysis against nr database in Genbank. The primer set (*aombb*-F: 5'-GAAATTCCCTCAATGCCACTTT-3' and *aombb*-R: 5'-TGTGATTTCCGGATCGACACT-3') and TaqMan probe (*aombb*-P: 5'-FAM-TGTGATTTCCGGATCGACACT-TAMRA-3') for quantitative real-time PCR were designed using Primer Express version 2.0 (Applied Biosystems, MA). Settings were put at default except for annealing temperature at 57°C and PCR product size between 50-150 bp. Based on *in silico* analysis, it was determined that the PCR target sequences were present only in one copy within the genome sequence of RU47. Specific fragments of the *aombb* (93 bp) gene were amplified in 25 µl reaction mixtures containing 1 µl DNA, 5µL GoTaq® Flexi buffer (1x), 0.2mM of each dNTP, 2.5 mM $MgCl_2$, 0.2 µM *aombb*-F and *aombb*-R primers, 0.6 U GoTaq® Flexi DNA polymerase (Promega Corporation, Madison, WI, USA), and 12.9 µl ultrapure H_2O. The PCR program was 95 °C for 5 min, and 35 cycles of 95 °C for 1 min, 54 °C for 30 s and 72 °C for 30 s, and then 72 °C for 5 min before cooling down to 4 °C. The amplified *aombb* fragment was subsequently cloned in *E. coli* DH5α using pGEM-T Easy Vector system (Promega Corporation, Madison, WI, USA) according to the manufacturer´s protocol. The pGEM-T Vector was extracted using GeneJET Plasmid Miniprep kit (Thermo Scientific, Lithuania) and used for serial dilutions to establish the standard for qPCR. The specific fragment of the *aombb* gene was amplified in 50 µl reaction mixtures containing 5 µl DNA (1:5 diluted in ultrapure H_2O), 1.25 U HotStart Taq DNA polymerase (New England Biolabs, Ipswich, MA, USA), 5 µl HotStart buffer (1x), 0.2 mM of each dNTP, 3.75 mM MgCl2, 0.1 mg/ml bovine serum albumin (Fermentas, Waltham, MA, USA), 0.1 µM *aombb* qPCR primers *aombb*-F and *aombb*-R and 0.1 µM TaqMan probe (*aombb*-P) (Eurofins Genomics, Luxembourg). Reactions were run for 10 min at 95°C and 40 cycles of 30 s at 95°C and 30 s at 54°C in a real-time PCR cycler (CFX Connect; Bio-Rad, Munich, Germany). Specificity for *Pseudomonas* sp. RU47 of the primer system in combination with the Taqman probe was tested with 20 reference strains and amplicons with the *aombb* primer system were only detected with genomic DNA from RU47 and RU47-rfp (mutant of strain *Pseudomonas* sp. RU47 with a cloned RFP-plasmid) strains (Table S1). However, even if testing *in silico* or with reference strains indicated specificity of the primer sets, the experimental designs should include non-inoculated controls that serve as a final confirmation of primer specificity.

Supplemental Figures

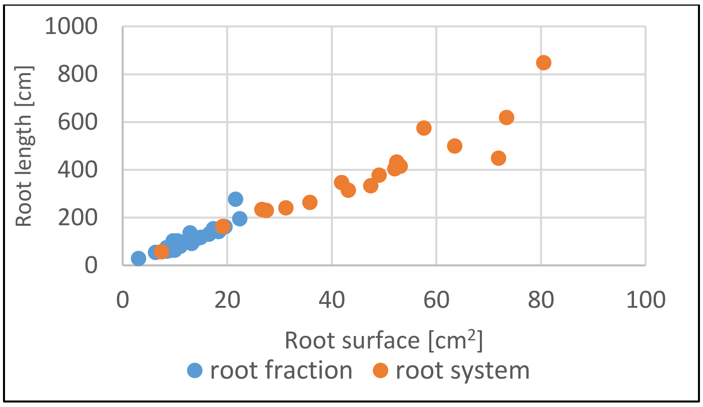

Fig. S1: Plotting of root length against root surface of subsampled roots (blue, n=24) and entire root systems (orange, n=18) of roots from apple M26 at 28 days post inoculation with sterile H_2O, *Bacillus velezensis* FZB42 or *Pseudomonas* sp. RU47 grown in ARD or grass soil.

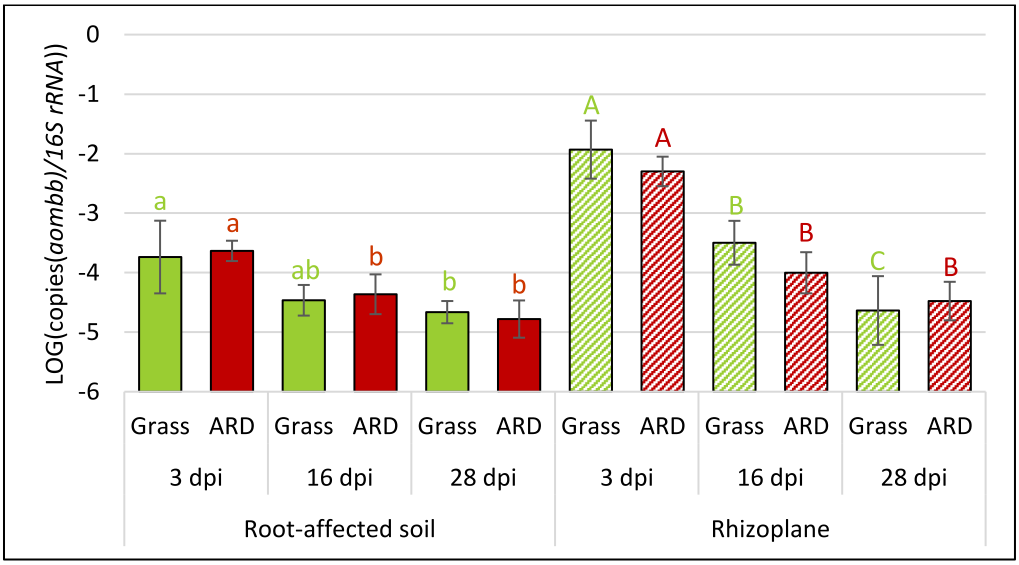

Fig. S2: Relative abundance of *Pseudomonas sp.* RU47 in microbial community DNA 3, 16 and 28 days after inoculation (dpi) in rhizoplane (RP, striped bars) and root-affected soil (RA, filled bars) from ARD- (red) and grass- (green) plots (Ellerhoop). Means of n=4 with standard deviation within a microhabitat (RP, minor letters; RA; capital letters) according to ANOVA followed by Tukeys' HSD test ($p<0.05$) are depicted. Asterisks indicate significant differences between two substrates (ARD vs. grass) within one microhabitat at one time point (3, 16 or 28 dpi) according to paired t-test ($p<0.05$: *).

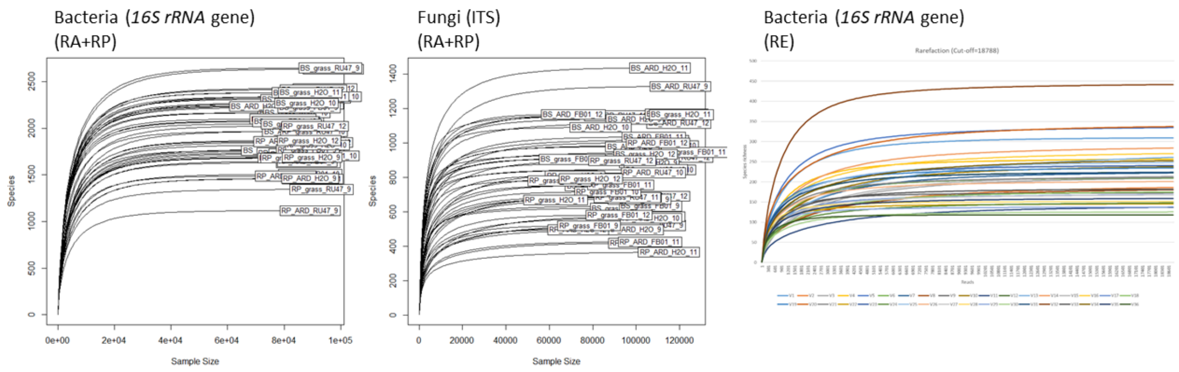

Fig. S3: Rarefaction curves based on ASVs derived from amplicon sequencing of the *16S rRNA* gene and ITS region of microbial community DNA from root-affected soil (RA), rhizoplane (RP) and root endosphere (RE, only *16S rRNA* gene).

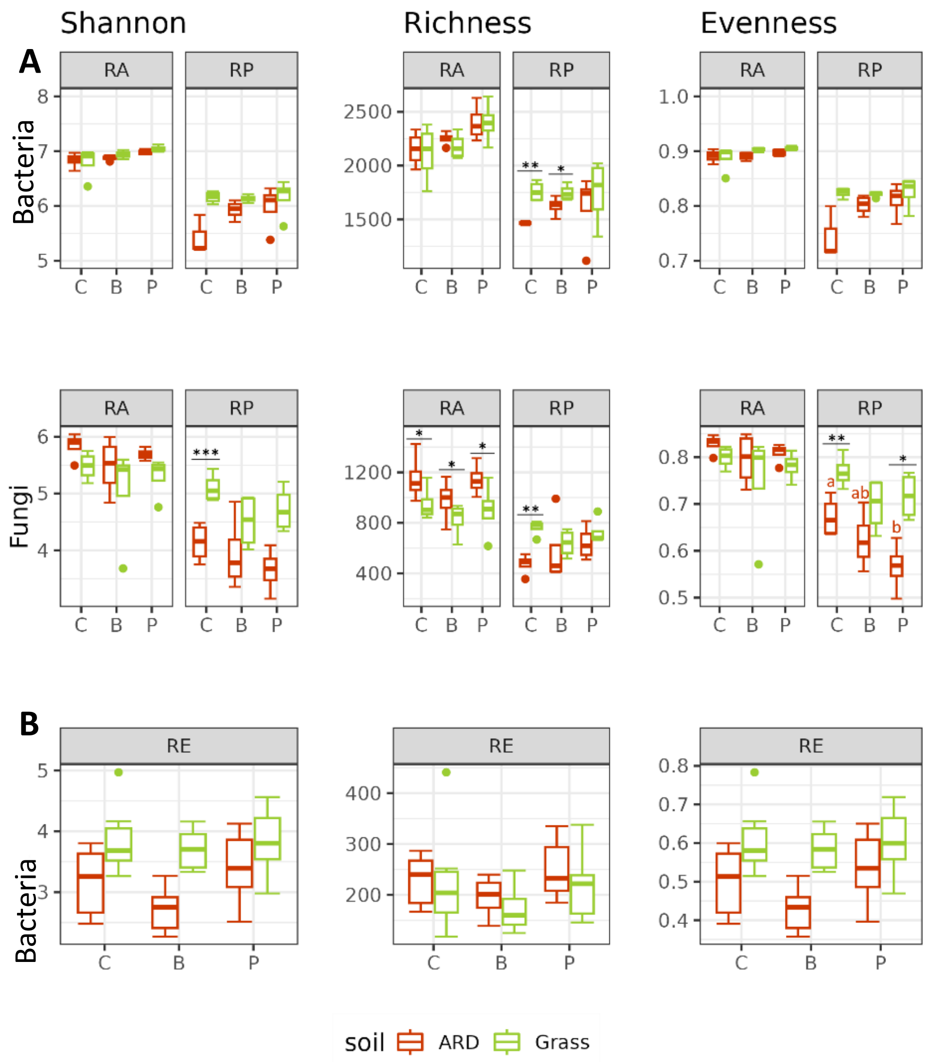

Fig. S4: α-diversity of apple M26 treated with sterile H_2O (C), *Bacillus velezensis* FZB42 (B) or *Pseudomonas* sp. RU47 (P) at 28 dpi. Different letters indicate significant differences ($p<0.05$) according to Kruskal-Wallis followed by Dunns' t-test within one soil (ARD or grass). Asterisks indicate significant differences in pairwise comparisons between ARD and grass soil of the same treatment (C, B or P; * p-value <0.05; ** p-value <0.01; *** p-value <0.001). **(A)** Bacterial (top) and fungal (bottom) communities in root-affected soil (RA) and rhizoplane (RP). Means of n=4 with standard error are depicted. **(B)** Bacterial communities in root endosphere (RE). Means of n=6 with standard error are depicted.

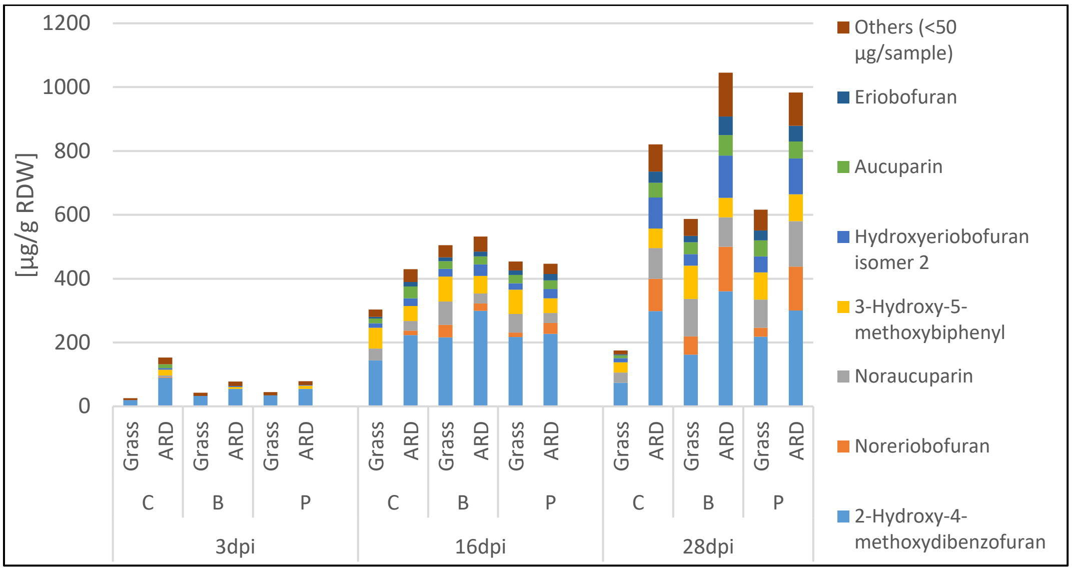

Fig. S5: Distibution of different phytoalexins in roots of apple M26 at 3, 16 and 28 days post inoculation (dpi) treated with sterile H_2O (C), *Bacillus velezensis* FZB42 (B) or *Pseudomonas* sp. RU47 (P) grown in ARD or grass soil. Means of n=4 are depicted. RDW: root dry weight

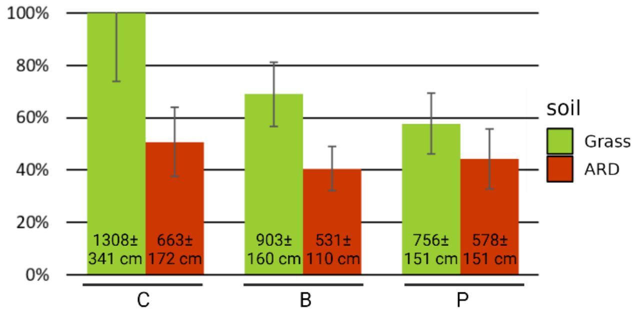

Fig. S6: Relative abundance of root length of roots from apple M26 at 28 days post inoculation (dpi) with sterile H_2O (C), *Bacillus velezensis* FZB42 (B) or *Pseudomonas* sp. RU47 (P) grown in ARD (red) or grass (green) soils. Means of n=7 including standard errors are depicted. Percentage values refer to root length in comparison to variant Grass_C. No significant differences in root length were observed.

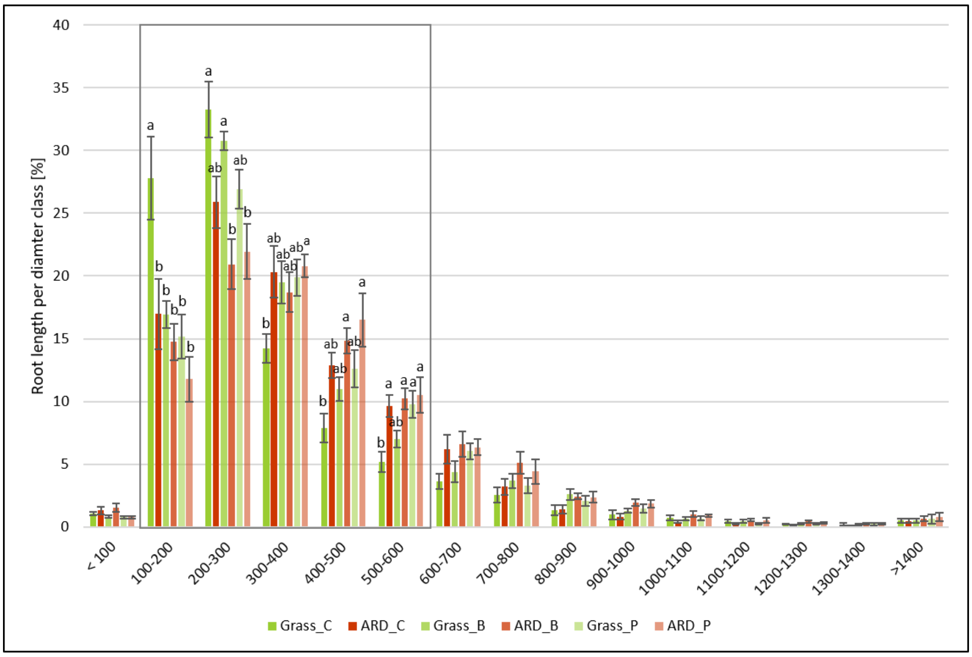

Fig. S7: Root length in different root diameter classes (class width 100 µm) 28 days after treatment with sterile H_2O (C), *Bacillus velezensis* FZB42 (B) or *Pseudomonas* sp. RU47 (P) grown on either ARD or grass soil. Data are derived from WinRhizo after destructive sampling of n=7 of which n=4 were subsamples and n=3 entire root systems. Different letters indicate significant differences according to ANOVA followed by Tukeys' HSD test.

Supplemental Tables

Table S1. Bacterial strains used for validation of strain specific qPCR primers and probe. +: strain was detected by qPCR, -: strain was not detected by qPCR and (number): cycle in which detection occurred.

	**Bacterial strain**	**References**	**Detection by qPCR**
1	*Pseudomonas* sp. RU47	Adesina et al., (2007)	**+ (18)**
2	*Pseudomonas* sp. RU47 *rfp*		**+ (20)**
3	*Pseudomonas fluorescens* R2f	Smit et al., (1991)	-
4	*Pseudomonas putida* KT2442	Nelson et al., (2002)	-
5	*Pseudomonas resinovorans* CA 10	Shintani et al., (2013)	-
6	*Pseudomonas aeruginosa* PST-1	Ogino et al., (1995)	-
7	*Pseudomonas aeruginosa* F-03	Fouhy et al., (2014)	-
8	*Pseudomonas fluorescens* NCIMB 10586	El-Sayed et al., (2003)	-
9	*Pseudomonas fluorescens* CHAO	Raaijmakers et al., (1997)	-
10	*Pseudomonas fluorescens* KS16	Adesina et al., (2009)	-
11	*Pseudomonas fluorescens* KS90	Adesina et al., (2009)	-
12	*Pseudomonas fulgida* KS70	Adesina et al., (2009)	-
13	*Pseudomonas fluorescens* KS74	Adesina et al., (2009)	-
14	*Pseudomonas fluorescens* KF36	Adesina et al., (2009)	-
15	*Pseudomonas koreensis* DSM 16610	Tvrzová et al., (2006)	-
16	*Pseudomonas jesseneii* DSM 17150	Ramírez-Bahena et al., (2014)	-
17	*Pseudomonas neruginosa* PA01	Stover et al., (2000)	-
18	*Pseudomonas migulae* D67	Jutkina et al., (2011)	-
19	*Pseudomonas savastanoi* pv. *gycinea*	Eltlbany et al., (2012)	-
20	*Pseudomonas* spp. DSMZ 13134	Buddrus-Schiemann et al., (2010)	-

Table S3: Relative abundance of 20 most abundant bacterial taxa in root-affected soil or rhizoplane of apple M26 grown in ARD or grass soil for 28 days. Plants were inoculated with sterile H_2O (C), *Bacillus velezensis* FB01 (B) or *Pseudomonas* sp. RU47 (P). Percentage of relative abundance of n=4 replicates is shown. Different minor letters indicate significant differences between treatments of the same soil: C, B and P in grass (green) or ARD (red). Capital letters indicate significant differences between grass and ARD soil within the three treatments: ARD_C vs. Grass_C (latin); ARD_B vs. Grass_B (italic); ARD_P vs. Grass_P (underlined). Significance of differences was tested based on pairwise comparison using generalized linear models in DeSeq2.

ASV	Taxon	Grass			ARD		
		C	B	P	C	B	P
		**Root-affected soil**					
ASV22	*Nitrospira*	0.57	0.63	0.74	0.79	1.02	1.21
ASV24	*Gaiellales*	0.73	0.69[A]	0.81[A]	0.62[a]	0.54 [cB]	0.61[bB]
ASV28	*Candidatus Udaeobacter*	0.59	0.62	0.64	0.49	0.54	0.54
ASV4	*Sphingomonadaceae*	0.73	0.41	0.44	0.70	0.76	0.76
ASV6	*Phenylobacterium*	0.63	0.37	0.45	0.54	0.57	0.72
ASV33	*Gaiella*	0.60	0.66	0.68	0.36[a]	0.26[c]	0.28[b]
ASV34	*Bacillus*	0.33	0.35	0.33[B]	0.50[b]	0.59[b]	0.67[aA]
ASV40	*Bacillus*	0.28	0.29	0.32	0.56	0.60	0.66
ASV32	*Bacillus*	0.35	0.33	0.37	0.47	0.44	0.52
ASV39	*Bacillus*	0.33[B]	0.29[B]	0.27[B]	0.52[bA]	0.49[bA]	0.57[aA]
ASV8	*Allo-Neo-Para-Rhizobium*	0.58	0.31	0.26[B]	0.45[ab]	0.48[a]	0.42[bA]
ASV21	*Sphingomonas*	0.45	0.44	0.41	0.37	0.59	0.55
ASV36	*Xanthobacteraceae*	0.41	0.46	0.43[A]	0.42[ab]	0.42[a]	0.38[bB]
ASV2	*Enterobacteriaceae*	0.63	0.83	0.39	1.39	1.74	0.99
ASV3	*Allo-Neo-Para-Rhizobium*	1.20[A]	0.64[B]	0.42[B]	1.10[abB]	1.03[aA]	0.81[bA]
ASV7	*Bradyrhizobium*	1.44[B]	1.56	1.20[B]	1.48[abA]	1.58[a]	1.23[bA]
ASV9	*Methyloligellaceae*	1.20	1.21	1.14	1.44	1.54	1.38
ASV1	*Streptomyces*	1.26	1.10	1.05[B]	1.80[a]	1.30[a]	1.25[bA]
ASV5	*Micrococcaceae*	1.68	1.52	1.22	1.11	1.20	0.86
ASV12	*Xanthobacteraceae*	1.11	1.19	1.07[A]	0.96[a]	0.80[a]	0.73[bB]
		**Rhizoplane**					
ASV10	*Enterobacteriaceae*	1.42	0.87[B]	0.81	3.67	1.82[A]	1.32
ASV8	*Allo-Neo-Para-Rhizobium*	1.17[B]	1.42	1.47	1.51[bA]	2.07[a]	2.07[a]
ASV13	*Para-Burkholderia-Caballeronia*	0.47[B]	0.58[B]	0.88	1.50[A]	2.16[A]	1.37
ASV26	*Sphingobium*	1.92[A]	0.97	0.67	0.85[B]	0.33	0.16
ASV23	*Novosphingobium*	0.49	0.80	0.33	1.11	1.10	0.42
ASV14	*Novosphingobium*	0.75[B]	0.83[B]	1.15	1.18[aA]	1.26[aA]	1.03[b]
ASV15	*Allo-Neo-Para-Rhizobium*	0.67	0.85	1.09	0.79[b]	1.26[b]	1.49[a]
ASV18	*Rhizobiaceae*	0.85	1.15	1.12	0.68[b]	0.95[ab]	1.31[a]
ASV16	*Novosphingobium*	1.07[A]	1.31	1.20	0.47[bB]	0.75[ab]	0.95[a]
ASV7	*Bradyrhizobium*	1.08	0.98	0.73	0.78	0.76	0.54
ASV5	*Micrococcaceae*	0.98[A]	0.87	0.81	0.77[aB]	0.69[b]	0.72[b]
ASV17	*Novosphingobium*	0.69	1.30	0.76	0.73	1.51	0.63
ASV20	*Sphingobium*	0.51	1.16[B]	0.65[B]	0.76[b]	1.33[aA]	0.90[aA]
ASV25	*Massilia*	0.48	0.77	0.67	0.58	0.94	0.96
ASV27	*Sphingobium*	0.62	1.01[A]	0.79[A]	0.42	0.65[B]	0.78[B]
ASV2	*Enterobacteriaceae*	4.20[B]	2.61	2.27	11.87[aA]	5.59[ab]	4.17[b]
ASV1	*Streptomyces*	4.75	5.83	5.21	2.48	2.70	3.69
ASV3	*Allo-Neo-Para-Rhizobium*	2.73	2.47	2.23	3.42	3.91	4.01
ASV4	*Sphingmonadaceae*	2.41	3.09	2.82	1.44[b]	2.18[ab]	2.27[a]
ASV6	*Phenylobacterium*	1.71[A]	1.89[A]	2.80	1.33[B]	1.51[B]	2.17

Table S4: Relative abundance of 20 most abundant fungal taxa in root-affected soil or rhizoplane of apple M26 grown in ARD or grass soil for 28 days. Plants were inoculated with sterile H_2O (C), *Bacillus velezensis* FB01 (B) or *Pseudomonas* sp. RU47 (P). Percentage of relative abundance of n=4 replicates is shown. Different minor letters indicate significant differences between treatments of the same soil: C, B and P in grass (green) or ARD (red). Capital letters indicate significant differences between grass and ARD soil within the three treatments: ARD_C vs. Grass_C (latin); ARD_B vs. Grass_B (italic); ARD_P vs. Grass_P (underlined). Significance of differences was tested based on pairwise comparison using generalized linear models in DeSeq2.

**ASV**	**Taxon**	**Grass**			**ARD**		
		**C**	**B**	**P**	**C**	**B**	**P**
		**Root-affected soil**					
ASV5	*Cladosporium*	5.70^{a}	3.52$^{\mathrm{b}A}$	4.91$^{\mathrm{b}\underline{\mathrm{A}}}$	0.21	0.20^{B}	0.47$^{\underline{\mathrm{B}}}$
ASV6	*Cladosporium*	5.33	3.26	5.01	0.18	0.21	0.49
ASV7	*Mortierella*	3.97	2.05	5.79	1.42	0.87	1.25
ASV8	*Mortierella*	3.52	1.95	5.85	1.28	0.78	1.32
ASV65	*Ascobolaceae*	0.15	0.38	0.28	1.69	0.37	0.65
ASV66	*Ascobolaceae*	0.10^{B}	0.33^{B}	0.24$^{\underline{\mathrm{B}}}$	1.80^{A}	0.36^{A}	0.64^{A}
ASV11	*Mortierella*	1.28	0.02	0.04	1.92	0.33	0.80
ASV15	*Mortierella*	1.05^{B}	0.02	0.03	1.63^{A}	0.63	0.90
ASV13	*Gibellulopsis*	1.38^{b}	1.00$^{\mathrm{c}B}$	2.13$^{\mathrm{a}\underline{\mathrm{B}}}$	2.56	1.29^{A}	2.33$^{\underline{\mathrm{A}}}$
ASV14	*Gibellulopsis*	1.29	0.97	2.06$^{\underline{\mathrm{B}}}$	2.32	1.24	2.45$^{\underline{\mathrm{A}}}$
ASV9	*Solicoccozyma*	1.16	0.88	1.95	1.64	1.23	2.10
ASV10	*Solicoccozyma*	1.34	0.89	1.83	1.74	1.16	1.99
ASV32	*Solicoccozyma*	0.72^{B}	0.44^{B}	1.34	0.93^{A}	0.68^{A}	1.29
ASV33	*Solicoccozyma*	0.79^{B}	0.39^{B}	1.28$^{\underline{\mathrm{A}}}$	1.02^{A}	0.62^{A}	1.18$^{\underline{\mathrm{B}}}$
ASV20	*Mortierella*	1.14	0.99	1.66	1.09	0.78	1.01
ASV21	*Mortierella*	0.92	0.90	1.68	1.03	0.71	1.08
ASV34	*Paraphaeosphaeria*	0.59	0.30	0.71	1.88	0.93	1.62
ASV35	*Paraphaeosphaeria*	0.56^{B}	0.28^{B}	0.74$^{\underline{\mathrm{B}}}$	1.76^{A}	0.89^{A}	1.54$^{\underline{\mathrm{A}}}$
ASV26	*Fusarium*	0.70^{B}	0.61	0.58	0.76^{A}	0.64	0.91
ASV30	*Fusarium*	0.73^{B}	0.53^{B}	0.55$^{\underline{\mathrm{B}}}$	0.79^{A}	0.58^{A}	0.82$^{\underline{\mathrm{A}}}$
		**Rhizoplane**					
ASV1	*Thelonectria*	1.93^{B}	0.84^{B}	4.99$^{\underline{\mathrm{B}}}$	12.63^{A}	9.90^{A}	21.40$^{\underline{\mathrm{A}}}$
ASV2	*Thelonectria*	2.05	0.90	4.74	12.83	10.16	20.34
ASV3	*Thelonectria*	2.90^{a}	1.48^{b}	5.96^{a}	3.31^{b}	5.35^{a}	3.68^{b}
ASV4	*Thelonectria*	2.93^{B}	1.43^{B}	6.14$^{\underline{\mathrm{A}}}$	3.40^{A}	4.68^{A}	3.86$^{\underline{\mathrm{B}}}$
ASV6	*Cladosporium*	5.04	3.31	3.50	0.19	0.10	0.14
ASV5	*Cladosporium*	5.17^{a}	3.60^{b}	3.31^{b}	0.19^{a}	0.08^{b}	0.12^{a}
ASV8	*Mortierella*	2.94	1.49	2.50	0.27	0.15	0.48
ASV7	*Mortierella*	3.24	1.52	2.25	0.25	0.16	0.42
ASV40	*Ilonectria*	0.07^{B}	0.03^{B}	0.04$^{\underline{\mathrm{B}}}$	2.94^{A}	1.28^{A}	0.83$^{\underline{\mathrm{A}}}$
ASV27	*Moesziomyces*	2.38^{A}	1.12^{A}	0.51$^{\underline{\mathrm{B}}}$	0.73^{B}	0.36^{B}	0.61$^{\underline{\mathrm{A}}}$
ASV28	*Moesziomyces*	2.37^{a}	1.23^{b}	0.45^{c}	0.75^{a}	0.30^{b}	0.52^{a}
ASV29	*Pseudogymnoascus*	0.12	0.96	0.98	1.58	0.92	2.04
ASV31	*Pseudogymnoascus*	0.20	0.60	0.93	1.73	0.93	1.93
ASV9	*Solicoccozyma*	1.18	0.62	2.56	1.01	0.35	0.82
ASV10	*Solicoccozyma*	1.24	0.63	2.33	1.03	0.35	0.73
ASV39	*Pseudogymnoascus*	0.54^{B}	0.29^{B}	1.44$^{\underline{\mathrm{B}}}$	0.83^{A}	0.56^{A}	1.51$^{\underline{\mathrm{A}}}$
ASV37	*Pseudogymnoascus*	0.60	0.26	1.36	0.92	0.59	1.31
ASV52	*Plectosphaerella*	0.00$^{\mathbf{b}}$	5.01$^{\mathrm{a}A}$	0.00^{b}	0.00^{b}	0.28$^{\mathrm{a}B}$	0.00^{b}
ASV12	*Thelonectria*	0.00	0.38	0.00	0.00	8.58	0.00
ASV25	*Thelonectria*	0.00^{b}	0.27$^{\mathrm{a}B}$	0.00^{b}	0.00^{b}	5.58$^{\mathrm{a}A}$	0.00^{b}

Table S5: Relative abundance of 20 most abundant bacterial taxa in root endosphere of apple M26 grown in ARD or grass soil for 28. Plants were inoculated with sterile H2O (C), *Bacillus velezensis* FB01 (B) or *Pseudomonas* sp. RU47 (P). Percentage of relative abundance of n=6 replicates is shown. Different minor letters indicate significant differences between treatments of the same soil: C, B and P in grass (green) or ARD (red). Capital letters indicate significant differences between grass and ARD soil within the three treatments: ARD_C vs. Grass_C (latin); ARD_B vs. Grass_B (italic); ARD_P vs. Grass_P (underlined). Significance of differences was tested based on pairwise comparison using generalized linear models in DeSeq2.

**ASV**	**Taxon**	**Grass**			**ARD**		
		**C**	**B**	**P**	**C**	**B**	**P**
		**Root endosphere**					
ASV2	*Allo-Neo-Para-Rhizobium*	11.87	10.55^{B}	9.87	26.63^{b}	39.84$^{\mathrm{a}\mathit{A}}$	28.60^{b}
ASV8	*Pseudomonas*	9.04^{B}	12.72^{A}	23.27	14.54^{aA}	6.22$^{\mathrm{b}\mathit{B}}$	11.94^{b}
ASV4	*Delftia*	10.37^{A}	17.41^{A}	9.98	0.01^{B}	0.16^{B}	0.00
ASV10	*Herbaspirillum*	8.09	1.91	2.33$^{\underline{\mathrm{A}}}$	5.26	11.06	0.60$^{\underline{\mathrm{B}}}$
ASV18	*Para-Burkholderia-Caballeronia*	2.32^{B}	2.88	6.15	12.62^{A}	4.89	10.49
ASV29	*Streptomyces*	4.54^{B}	3.29^{B}	2.32$^{\underline{\mathrm{B}}}$	9.35^{A}	10.99^{A}	6.86$^{\underline{\mathrm{A}}}$
ASV64	*Rhizobacter*	0.03	0.21	0.09$^{\underline{\mathrm{B}}}$	1.31	0.16	8.57$^{\underline{\mathrm{A}}}$
ASV48	*Raoultella*	0.00 ^{b}	2.10^{a}	1.58^{a}	1.52	0.57	2.80
ASV129	*Flavobacterium*	0.63	0.45	0.49	2.24	0.49	3.39
ASV88	*Asticcacaulis*	1.43^{A}	0.99^{A}	2.13$^{\underline{\mathrm{A}}}$	0.66^{cB}	0.69$^{\mathrm{a}\mathit{B}}$	0.68$^{\mathrm{b}\underline{\mathrm{B}}}$
ASV47	*Phenylobacterium*	2.99	0.76	1.22	0.54	0.21	0.58
ASV97	*Sphingomonas*	2.51	4.96	1.50	0.23	0.71	0.39
ASV11	*Pedobacter*	2.43	3.99	2.81	0.06	0.11	0.42
ASV9	*Bosea*	2.46^{bA}	4.11^{a}	2.51^{a}	0.02^{B}	0.07	0.05
ASV39	*Rhodanobacter*	2.60	0.71	2.29	2.65	3.87	0.86
ASV119	*Massilia*	2.22	0.93	0.77	1.16	3.55	1.41
ASV40	*Bordetella*	1.24	0.86	1.63	1.08	1.94	2.71
ASV31	*Novosphingobium*	5.67	3.73	6.27	2.95	1.66	1.90
ASV56	*Sphingobium*	1.80^{B}	3.17^{A}	1.69$^{\underline{\mathrm{A}}}$	3.94^{A}	1.26^{B}	0.89$^{\underline{\mathrm{B}}}$
ASV42	*Acidovorax*	4.98^{aA}	2.15$^{\mathrm{a}\ \mathit{A}}$	1.46^{b}	1.77^{B}	0.32^{B}	1.85
